# Stringent homology-based prediction of *H. sapiens-M. tuberculosis* H37Rv protein-protein interactions

**DOI:** 10.1186/1745-6150-9-5

**Published:** 2014-04-08

**Authors:** Hufeng Zhou, Shangzhi Gao, Nam Ninh Nguyen, Mengyuan Fan, Jingjing Jin, Bing Liu, Liang Zhao, Geng Xiong, Min Tan, Shijun Li, Limsoon Wong

**Affiliations:** 1 NUS Graduate School for Integrative Sciences & Engineering, National University of Singapore, Singapore, Singapore; 2 School of Computing, National University of Singapore, Singapore, Singapore; 3 Department of Medicine, Brigham and Women’s Hospital, Boston, USA; 4 Department of Microbiology and Immunobiology, Harvard University, Cambridge, USA; 5 Department of Environmental Health, Harvard School of Public Health, Harvard University, Cambridge, USA; 6 Computer Science Department, Carnegie Mellon University, Pittsburgh, PA 15213, USA; 7 Bioinformatics Research Center, & School of Computer Engineering, Nanyang Technological University, Singapore, Singapore

## Abstract

**Background:**

*H. sapiens*-*M. tuberculosis* H37Rv protein-protein interaction (PPI) data are essential for understanding the infection mechanism of the formidable pathogen *M. tuberculosis* H37Rv. Computational prediction is an important strategy to fill the gap in experimental *H. sapiens*-*M. tuberculosis* H37Rv PPI data. Homology-based prediction is frequently used in predicting both intra-species and inter-species PPIs. However, some limitations are not properly resolved in several published works that predict eukaryote-prokaryote inter-species PPIs using intra-species template PPIs.

**Results:**

We develop a stringent homology-based prediction approach by taking into account (i) differences between eukaryotic and prokaryotic proteins and (ii) differences between inter-species and intra-species PPI interfaces. We compare our stringent homology-based approach to a conventional homology-based approach for predicting host-pathogen PPIs, based on cellular compartment distribution analysis, disease gene list enrichment analysis, pathway enrichment analysis and functional category enrichment analysis. These analyses support the validity of our prediction result, and clearly show that our approach has better performance in predicting *H. sapiens*-*M. tuberculosis* H37Rv PPIs. Using our stringent homology-based approach, we have predicted a set of highly plausible *H. sapiens*-*M. tuberculosis* H37Rv PPIs which might be useful for many of related studies. Based on our analysis of the *H. sapiens*-*M. tuberculosis* H37Rv PPI network predicted by our stringent homology-based approach, we have discovered several interesting properties which are reported here for the first time. We find that both host proteins and pathogen proteins involved in the host-pathogen PPIs tend to be hubs in their own intra-species PPI network. Also, both host and pathogen proteins involved in host-pathogen PPIs tend to have longer primary sequence, tend to have more domains, tend to be more hydrophilic, etc. And the protein domains from both host and pathogen proteins involved in host-pathogen PPIs tend to have lower charge, and tend to be more hydrophilic.

**Conclusions:**

Our stringent homology-based prediction approach provides a better strategy in predicting PPIs between eukaryotic hosts and prokaryotic pathogens than a conventional homology-based approach. The properties we have observed from the predicted *H. sapiens*-*M. tuberculosis* H37Rv PPI network are useful for understanding inter-species host-pathogen PPI networks and provide novel insights for host-pathogen interaction studies.

**Reviewers:**

This article was reviewed by Michael Gromiha, Narayanaswamy Srinivasan and Thomas Dandekar.

## Background

Tuberculosis is a major infectious disease which causes about 2 million deaths each year. The causative agent of this disease—*M. tuberculosis*—infects around one-third of the world’s population [1,2]. Tuberculosis is also the most common opportunistic infection in HIV-infected patients and one of the most common causes of death among people dying with AIDS [3,4].

Host-pathogen PPIs are very important for understanding infection mechanisms. However, such inter-species PPIs are not readily available in many host-pathogen systems. Several computational approaches have been developed to predict host-pathogen PPIs, including approaches based on homology, interacting domain/motif, structure, and even machine learning [5]. Homology-based approaches are the conventional way of predicting both intra-species and inter-species PPIs, with the assumption that the interaction between a pair of proteins in one species is likely to be conserved in related species [6]. They are also among the most frequently used methods in predicting host-pathogen PPIs, either being used alone [7-10] or in combination with other methods [11].

Current homology-based approaches generally transfer intra-species PPIs to predict host-pathogen PPIs. There are several limitations and concerns that have yet to be addressed. For example, (i) the protein-protein interaction interfaces between intra-species PPI and inter-species PPI are not exactly the same [12]; (ii) the differences between prokaryotic and eukaryotic proteins are not considered. Therefore, the performance of conventional homology-based host-pathogen PPI prediction approaches is rather limited [7-10]. In fact, most of these published works lack stringent verification. Thus, the accuracy of conventional homology-based approaches in predicting host-pathogen PPI is largely unknown.

In this work, we develop a novel homology-based approach for predicting the *H. sapiens-M. tuberculosis* H37Rv PPIs by specifically transferring the eukaryote-prokaryote PPIs from an experimental human-bacteria template PPI dataset. Moreover, we adopt a more stringent method in identifying homologs between species by taking genomic context into account. This prediction approach specifically addresses the limitations of conventional homology-based approaches. In this work, we focus on direct physical protein-protein interactions; therefore all the PPIs mentioned in this work are direct physical protein-protein interactions.

Cellular compartment distribution analysis, disease-related enrichment analysis, pathway enrichment analysis, and functional category enrichment analysis show that our predicted *H. sapiens-M. tuberculosis* H37Rv PPI dataset has good quality. These analyses also demonstrate that our stringent homology-based approach has much better performance than a conventional homologybased approach. Therefore this stringent homology-based approach can be used for predicting host-pathogen PPIs in a variety of different eukaryote-prokaryote host-pathogen systems.

Based on primary sequence analysis and topological analysis of the predicted host-pathogen protein-protein interaction network (PPIN), we discover some interesting properties of both pathogen and host proteins participating in host-pathogen PPIs, including the tendency to be hubs in the intra-species PPIN, tendency to have smaller average shortest path length, tendency to be more hydrophilic, tendency to have longer sequences and more domains. Furthermore, the domains in the proteins involved in host-pathogen PPIN tend to have lower charge and tend to be more hydrophilic in comparison with other proteins in the intra-species PPIN.

## Methods

Our stringent homology-based approach for predicting host-pathogen (*H. sapiens-M. tuberculosis* H37Rv) PPIs specifically transfers eukaryote-prokaryote (human-bacteria) PPIs from the PATRIC database [13]. Cellular compartment distribution analysis, disease-related enrichment analysis, pathway enrichment analysis, and functional category enrichment analysis strongly support our prediction results and show that the predicted PPIs correspond to the *M. tuberculosis* H37Rv infection process.

In a control study, we use a conventional homology-based approach to predict possible host-pathogen (*H. sapiens-M. tuberculosis* H37Rv) PPIs. The same distribution and enrichment analyses are conducted on both results predicted by our stringent approach and the conventional approach. The comparison shows that our stringent homology-based approach has better performance in predicting more relevant and meaningful host-pathogen PPI than the conventional approach.

We further analyze some of the basic sequence properties of proteins involved in the host-pathogen PPIN comparing with the counterparts involved in intra-species PPIN by examining the sequences, domains, hydrophobicity scales, domain interaction degrees, electronic charge, etc. We also perform topological analysis to illuminate the intra-species topological properties of both the host and pathogen proteins involved in the predicted *H. sapiens-M. tuberculosis* H37Rv PPIN.

### Prediction of host-pathogen PPI networks

**Figure 1:**
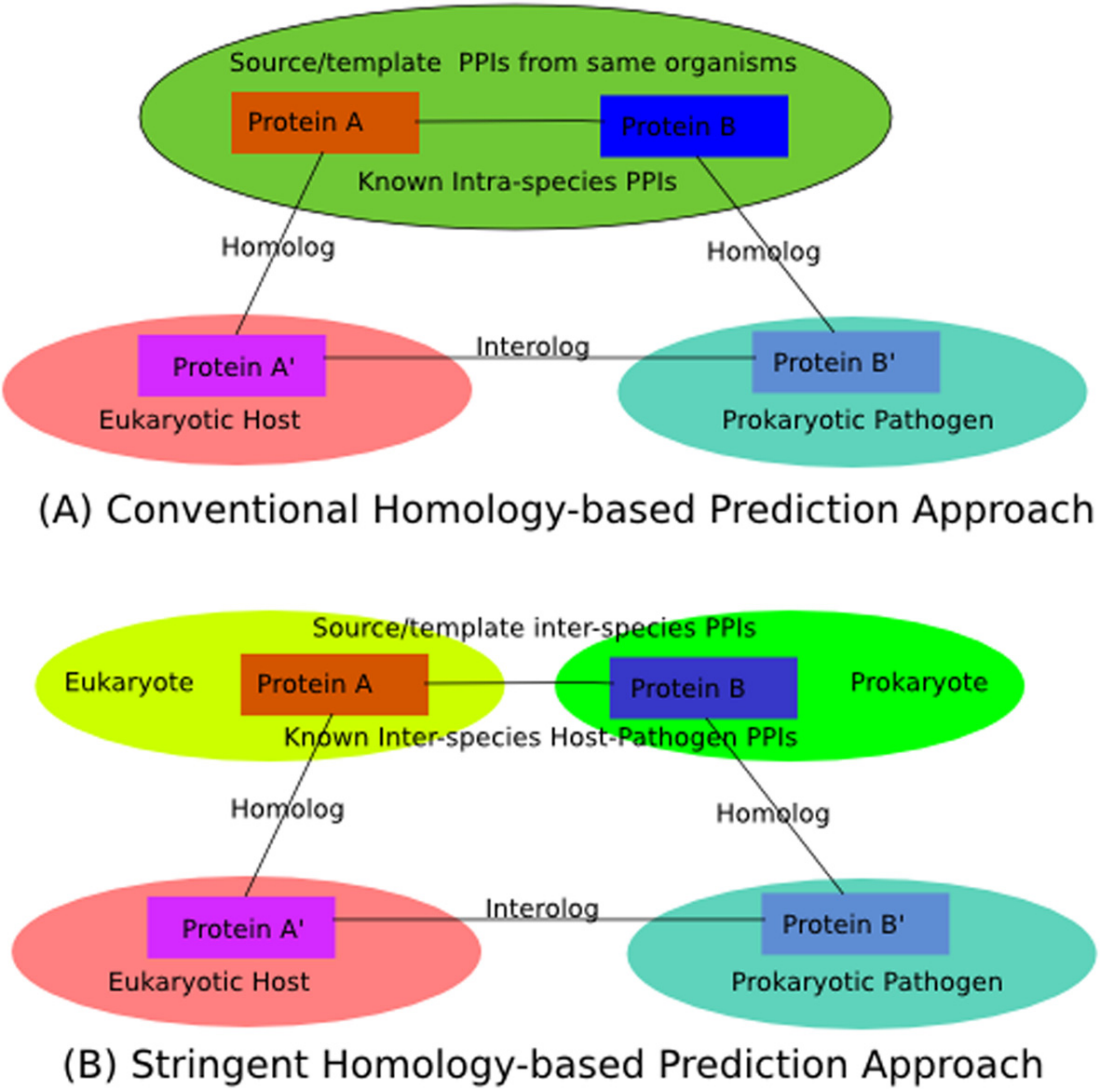
**Representation of homology-based prediction approach.** Representation of **(A)** the conventional homology-based prediction approach and **(B) ** the stringent homology-based prediction approach adopted in this study.

Conventional homology-based approaches generally transfer intra-species PPIs to predict host-pathogen PPIs. That is, if a protein X in the host and a protein Y in the pathogen are respectively homologous to a pair of proteins X’ and Y’ which are known to interact in a third species, X and Y are predicted to interact. In contrast, our stringent homology-based approach specifically transfers eukaryote-prokaryote inter-species PPIs to predict host-pathogen PPIs. Specifically, if a protein X in a eukaryotic host is known to interact with a protein Y’ in a prokaryote species, and Y’ is homologous to a protein Y in a prokaryotic pathogen, then we predict X and Y to interact. Moreover, to more accurately determine homologous proteins with conserved interactions, we use a homolog matching method that takes genomic context into consideration. This stringent homology-based approach takes the followings into account: (i) the interface between intra- and inter-species PPI are not exactly the same [12]; (ii) the differences between prokaryotic and eukaryotic proteins are also very obvious (post-transcriptional modifications, structures, signal peptide, cleavage site). Figure 1 shows differences between (a) a conventional homology-based prediction approach and (b) our approach.

For the stringent homology-based approach, we collect from the PATRIC database [13] the template eukaryote-prokaryote human-bacteria PPIs and the genome sequences and gene feature files of relevant bacteria strains.

The list of bacteria strains in the PATRIC database [13] relevant to our study are *Bacillus anthracis str.* A2012, *Bacillus anthracis str.* Ames Ancestor, *Bacillus anthracis str.* Ames, *Bacillus anthracis str.* Sterne, *Francisella tularensis subsp tularensis* MA00-2987, *Francisella tularensis subsp tularensis* SCHU S4, *Shigella flexneri 2a str.* 301, *Yersinia pestis biovar Microtus* str. 91001, *Yersinia pestis* CO92, and *Yersinia pestis* KIM. These 10 major strains of bacteria cover 7120 PPIs in the PATRIC database, constituting 99% of the total PPIs contained in the database (data downloaded on April 3, 2012). The dataset collected above (PPIs between human and 10 major bacteria species) are the most abundant source eukaryote-prokaryote inter-species PPIs.

Our stringent homology-based prediction strategy works as follows. If a human protein A is known to interact with a bacteria protein B in a template PPI (we call this template PPI a supporting template PPI), and the bacteria protein B has a homolog B’ identified in *M.tuberculosis* H37Rv, then we predict that the human protein A and the *M.tuberculosis* H37Rv protein B’ also interact with each other.

We count the number of supporting template PPIs as the “consensus score” of each predicted *H. sapiens-M. tuberculosis* H37Rv PPI. This serves as one of the important parameters for evaluating how likely the predicted PPI is real compared with the rest of the predicted PPIs.

**Figure 2:**
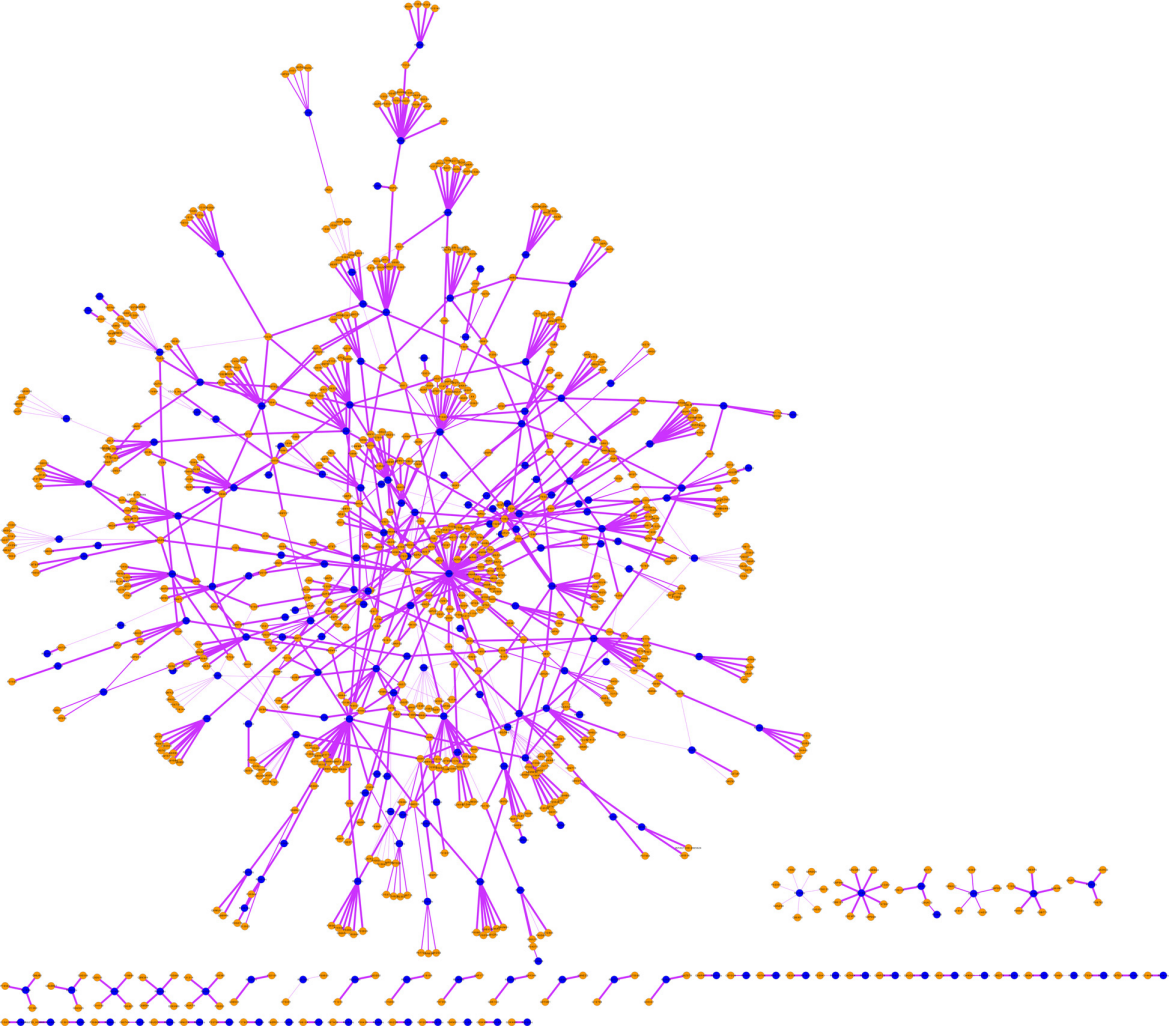
**Visualization of the predicted *H. sapiens-M. tuberculosis *H37Rv PPI network.** The blue dots are *M. tuberculosis* H37Rv proteins, while the orange dots are *H. sapiens* proteins. The “thickness” of an edge corresponds to the “consensus score” of the predicted *H. sapiens-M. tuberculosis* H37Rv PPI, the thicker the edge the larger of the “consensus score”.

Using the stringent prediction approach as described above, we have predicted 1005 *H. sapiens-M. tuberculosis* H37Rv PPIs (Additional file 1). We visualize the predicted network using Cytoscape [14] in Figure 2.

We also predict host-pathogen PPIs using a conventional homology-based approach as a control experiment. Different from the stringent homology-based approach, the conventional homology-based approach uses template intra-species *H. sapiens* physical PPIs collected from three major PPI databases, MINT [15], BioGRID [16], and IntAct [17]. All together 73251 *H. sapiens* physical PPIs are collected(data was downloaded on November 10, 2011). To predict *H. sapiens–M. tuberculosis* H37Rv PPIs using the conventional homology-based approach, we identify homologs between *H. sapiens* and *M. tuberculosis* H37Rv, and then transfer the intra-species *H. sapiens* PPIs to predict the inter-species *H. sapiens–M. tuberculosis* H37Rv PPIs.

The conventional homology-based prediction strategy uses different template PPIs for the prediction: if a human protein A interacts with a human protein B in a template PPI, and the human protein B has a homolog B’ identified in *M.tuberculosis* H37Rv, then it predicts that the human protein A and the *M.tuberculosis* H37Rv protein B’ interact with each other. Using the conventional homology-based prediction approach as described above, we have predicted 326 *H. sapiens-M. tuberculosis* H37Rv PPIs.

To identify the homologs between *M.tuberculosis* H37Rv and the 10 bacteria (in our stringent approach) and also the between *M.tuberculosis* H37Rv and *H. sapiens* (in the conventional approach), we use the BBH-LS algorithm which computes positional homologs based on both sequence and gene context similarity [18]. BBH-LS is an effective and simple method to identify the positional homologs from the comparative analysis of two genomes. It integrates sequence similarity and gene context similarity in order to identify accurate orthologs [18]. This method applies the bidirectional-best-hit heuristic to a combination of sequence similarity and gene context similarity scores [18]. When BBH-LS was applied to the human, mouse, and rat genomes, it produced the best results when using both sequence and gene context information equally. Compared to other classic algorithms (like MSOAR2), BBH-LS can identify more homologs with less false positives [18]. BBH-LS is considered to be a more accurate way of identifying homologs than other approaches which do not consider both the sequence and gene context similarity. The BBH-LS strength threshold *β* in this work is set as 0.01.

### Cellular compartment distribution of *H. sapiens* proteins targeted by the predicted host-pathogen PPI

The cellular compartment of the *H. sapiens* proteins targeted by the predicted host-pathogen PPIs are an important indicator of the quality of predicted PPIs. If the targeted *H. sapiens* proteins are located in cellular compartments that are very relevant to the pathogen’s infection or are very likely to be involved in interactions with the pathogen, then the result supports the host-pathogen predictions.

**Figure 3:**
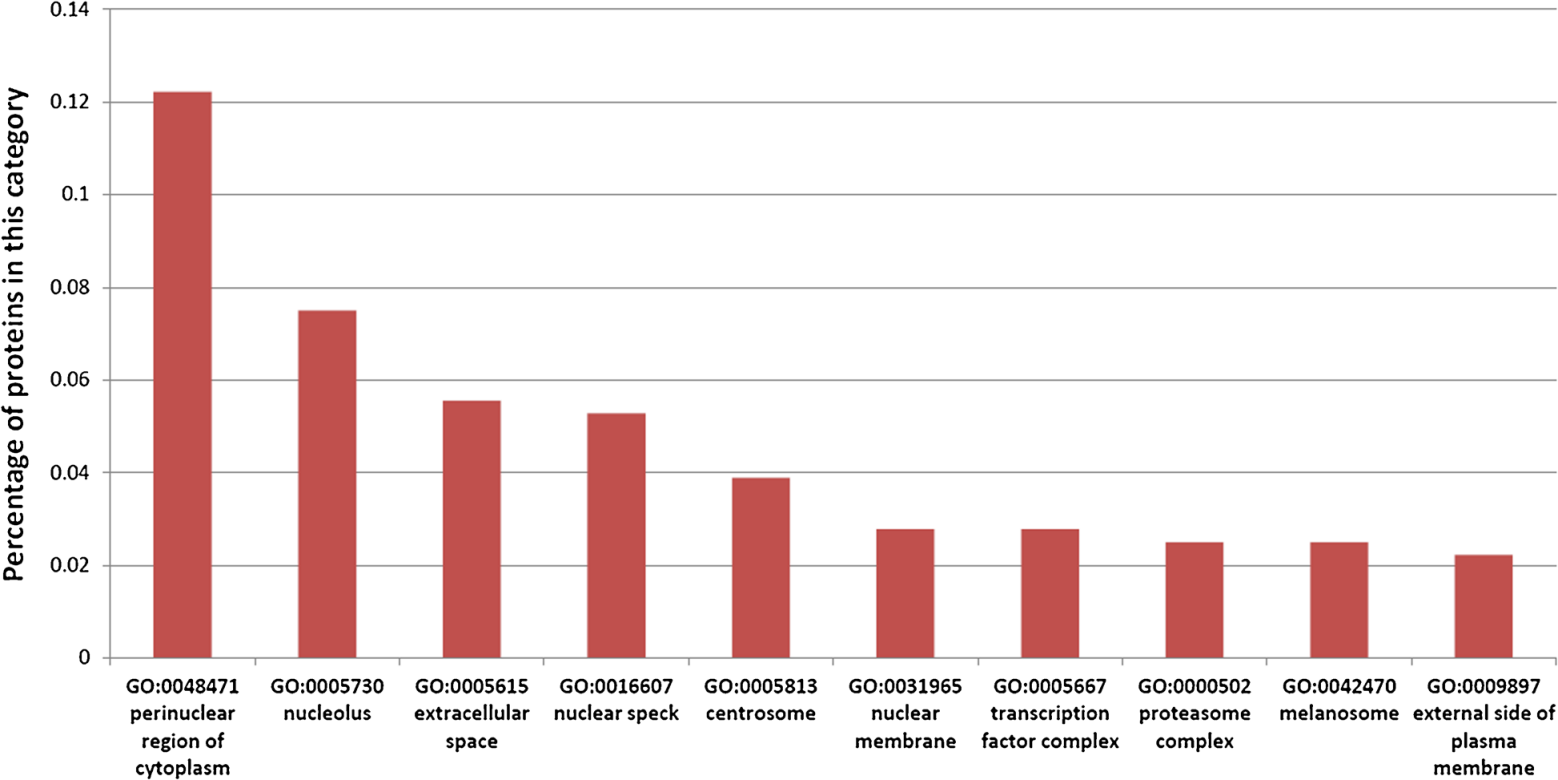
**Cellular compartment distribution of *H. sapiens *proteins targeted by the stringent homology-based approach predicted host-pathogen PPIs.** Cellular compartment distribution of *H. sapiens* proteins targeted by the stringent homology-based approach predicted host-pathogen PPIs (Top 10 cellular compartments).

**Figure 4:**
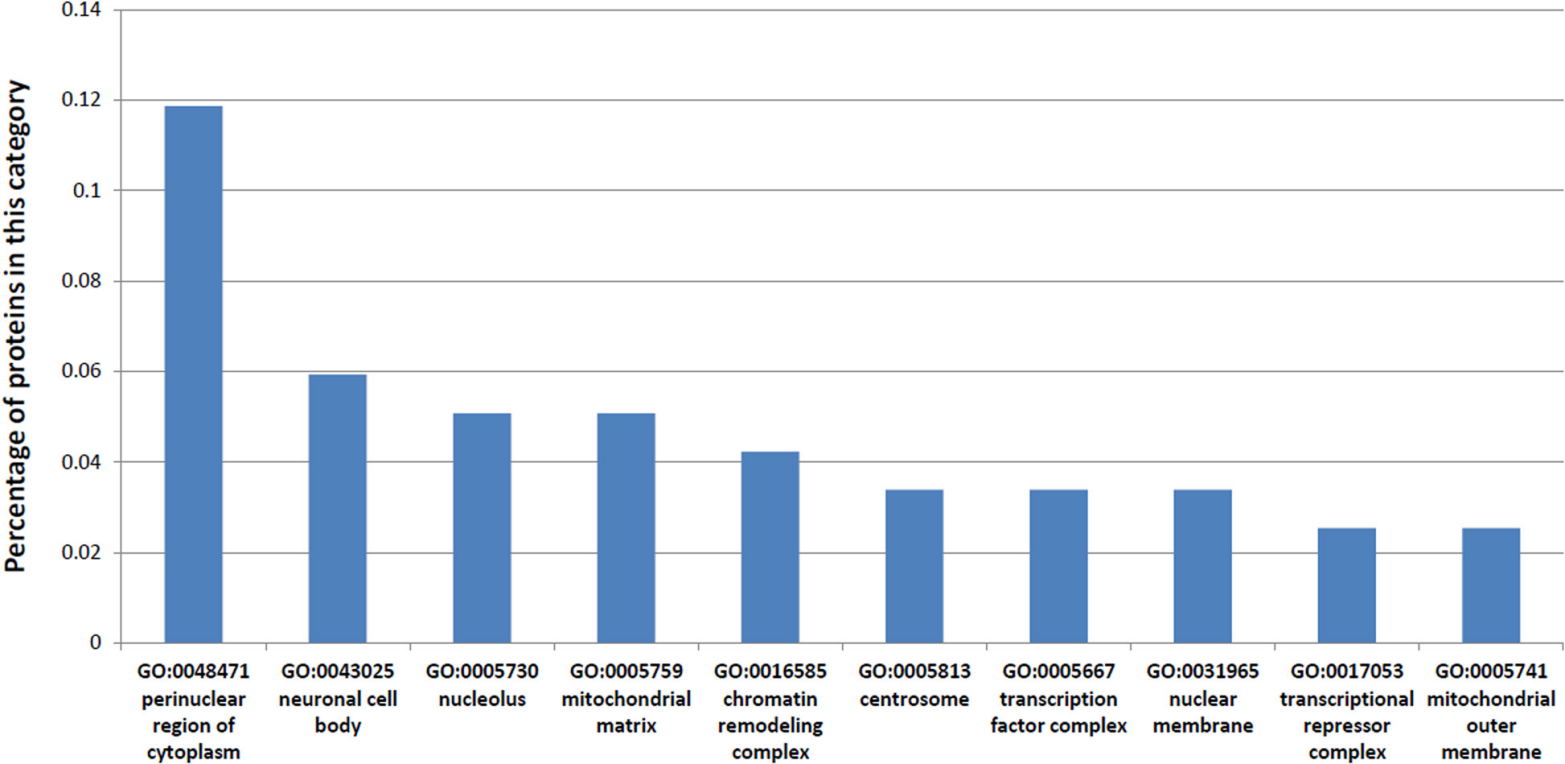
**Cellular compartment distribution of *H. sapiens *proteins targeted by the conventional homology-based approach predicted host-pathogen PPIs.** Cellular compartment distribution of *H. sapiens* proteins targeted by the conventional homology-based approach predicted host-pathogen PPIs (Top 10 Cellular Compartments).

Gene Ontology (Cellular Compartment, CC) is one of the most comprehensive annotations for human proteins. Thus, we use it in our analysis. However, as the Gene Ontology is hierarchical, CC terms at the top levels may have more proteins annotated with them, while terms on lower levels may have less proteins annotated with them. Therefore, we only use informative CC terms for our analysis. An informative CC term is defined here to be a term that has at least 90 proteins annotated with it, but each of its child terms has less than 90 proteins annotated with it. The cellular compartment distribution tells how many proteins(and the percentage) in the datasets that fall into each cellular compartment. We choose the top 10 most frequently located cellular compartments of the *H. sapiens* proteins that are targeted by the stringent and the conventional homology-based prediction approaches. The results are shown in Table 1, Figure 3 and Figure 4.

**Table 1:** **Cellular compartment distribution of *H. sapiens *** **proteins targeted by the predicted host-pathogen PPIs**

**Cellular compartment**	**Percentage(%)**	**No. of**
		**proteins**
**(a)**		
GO:0048471 perinuclear region of cytoplasm	12.2	44
GO:0005730 nucleolus	7.50	27
GO:0005615 extracellular space	5.56	20
GO:0016607 nuclear speck	5.28	19
GO:0005813 centrosome	3.89	14
GO:0031965 nuclear membrane	2.78	10
GO:0005667 transcription factor complex	2.78	10
GO:0000502 proteasome complex	2.50	9
GO:0042470 melanosome	2.50	9
GO:0009897 external side of plasma membrane	2.22	8
**(b)**		
GO:0048471 perinuclear region of cytoplasm	11.9	14
GO:0043025 neuronal cell body	5.93	7
GO:0005730 nucleolus	5.08	6
GO:0005759 mitochondrial matrix	5.08	6
GO:0016585 chromatin remodeling complex	4.24	5
GO:0005813 centrosome	3.39	4
GO:0005667 transcription factor complex	3.39	4
GO:0031965 nuclear membrane	3.39	4
GO:0017053 transcriptional repressor complex	2.54	3
GO:0005741 mitochondrial outer membrane	2.54	3

This table summarizes top 10 most frequent cellular compartments where the *H. sapiens* proteins (targeted by the predicted host-pathogen PPIs) likely to be located in.

(a) is cellular compartment distribution of *H. sapiens * proteins targeted by the stringent homology-based approach predicted host-pathogen PPIs (Top 10 cellular compartments).

(b) is cellular compartment distribution of *H. sapiens* proteins targeted by the conventional homology-based approach predicted host-pathogen PPIs (Top 10 cellular compartments).

### Disease-related enrichment analysis of proteins involved in host-pathogen PPIs

Currently large-scale high-quality experimental *H. sapiens–M. tuberculosis* H37Rv PPIs are not readily available. Therefore a gold standard PPI dataset for assessing the predicted *H. sapiens–M. tuberculosis* H37Rv PPIs is not possible at the moment. However, there are several studies that examine *H. sapiens* gene expression profiles during *M. tuberculosis* H37Rv infection and treatment [19,20].

We obtain several *H. sapiens* gene lists related to *M. tuberculosis* H37Rv infection and treatment from two studies [19,20]. Chaussabel *et al.*[20] identified the unique gene expression profiles of human macrophages and dendritic cells responses to phylogenetically distinct parasites, including *M. tuberculosis* H37Rv. We name this gene list “Macrophages and dendritic differentially expressed genes”; it contains 1531 differentially expressed *H. sapiens* genes. In another study, Cliff *et al.*[19] identified several lists of blood gene expression profiles of tuberculosis treatment in different phases. Genes differentially expressed between diagnosis and week 1 of treatment are called “Early Changers” [19], comprising 470 differentially expressed *H. sapiens* genes. Genes differentially expressed between week 4 and week 26 of treatment are called “Late Changers” [19], comprising 327 differentially expressed *H. sapiens* genes. Genes which maintained a consistent pattern of change of gene expression and did not revert are called “Consistent Changers” [19], comprising 406 differentially expressed *H. sapiens* genes.

Monocyte-derived dendritic cells and macrophages generated *in vitro* from the same individual blood donors were exposed to pathogens(*M. tuberculosis*), and gene expression profiles were assessed by microarray analysis in the work of Chaussabel *et al.*[20]. The genes differentially expressed during the exposure to pathogens are consistent with the concept that antigen-presenting cells have specific genes for use in the response to pathogens like *M. tuberculosis*[20]. Therefore the list of genes differentially expressed when the dendritic cells and macrophages are exposed to *M. tuberculosis* may have high possibility of involving in *H. sapiens–M. tuberculosis* H37Rv PPIs.

In the work of Cliff *et al.ex vivo* blood samples were collected from 27 first-episode pulmonary tuberculosis patients prior to starting standard therapy and after 1, 2, 4, and 26 weeks of successful treatment. Genome-wide gene expression profiles were obtained from *ex vivo* blood samples, the differentially expressed genes in different phases are called Early Changers, Late Changers and Constant Changers. The fast initial down-regulation of expression of inflammatory mediators coincided with rapid killing of actively dividing bacilli, whereas slower delayed changes occurred as drugs acted on dormant bacilli and coincided with lung pathology resolution [19]. As the drugs are working on killing the bacilli (*M. tuberculosis*), the differentially expressed genes at different phases correspond to the response to different groups of *M. tuberculosis*(actively dividing bacilli, dormant bacilli, etc.). These disease gene lists have also been used in assessments of predicted host-pathogen PPIs in other studies [21]. These lists of differentially expressed genes form our reference disease-related gene lists. We conduct, against these disease-related gene lists, the enrichment (over-representation) analysis of the *H. sapiens* proteins involved in *H. sapiens–M. tuberculosis* H37Rv PPIs predicted by our stringent homology-based approach and by the conventional homology-based approach. The enrichment analysis uses the hypergeometric test. The results are given in Table 2.

**Table 2:** **Disease-related enrichment analysis of *H. sapiens *** **proteins involved in host-pathogen PPIs**

**Gene list**	**Overlap**	**p-value**
** (a)**		
Early Changers	32	1.022E-10
Late Changers	31	3.785E-14
Consistent Changers	35	1.500E-14
Early and Late Changers	56	6.996E-21
Early and Consistent Changers	49	3.721E-18
Consistent and Late Changers	42	1.499E-16
Macrophages and dendritic differentially expressed genes	107	2.097E-34
** (b)**		
Early Changers	6	3.08E-02
Late Changers	6	6.11E-03
Consistent Changers	8	1.04E-03
Early and Late Changers	10	2.94E-03
Early and Consistent Changers	9	4.30E-03
Consistent and Late Changers	9	1.07E-03
Macrophages and dendritic differentially expressed genes	35	5.23E-14

This table summarizes *H. sapiens* proteins’ (involved in the predicted host-pathogen PPIs) enrichment (over-representation) in *M. tuberculosis* H37Rv infection and treatment-related differentially expressed gene lists.

(a) is enrichment analysis results from the stringent homology-based approach.

(b) is enrichment analysis results from the conventional homology-based approach.

### Functional enrichment analysis of proteins involved in host-pathogen PPIs

Functional enrichment analysis is very important for identifying the functional relevance of the proteins involved in the host-pathogen PPIs. The presence of enriched (over-represented) functional categories that are closely related to pathogen infection, immune response, etc. serves as further support for the validity of the prediction results.

The Gene Ontology (Molecular Function, MF) is one of the most comprehensive functional categories annotation. Therefore we conduct MF term enrichment analysis on the *H. sapiens* proteins involved in the predicted *H. sapiens-M. tuberculosis* H37Rv PPIs.

In this work, we use the DAVID database [22] for the GO term enrichment analysis on the *H. sapiens* proteins involved in host-pathogen PPIs predicted by our stringent homology-based approach and the conventional homology-based approach. Representative results (significantly enriched level 5 MF terms, threshold “count >2, p-value <0.01”) are shown in Table 3, and complete results can be found in Additional file 2 (threshold “count >2, p-value <0.1”).

**Table 3:** **GO term enrichment analyses of *H. sapiens *** **proteins involved in the predicted host-pathogen PPI dataset**

**GO terms**	**p-value**
** (a)**	
GO:0051015 actin filament binding	6.12E-5
GO:0010843 promoter binding	5.76E-4
GO:0003713 transcription coactivator activity	7.18E-4
GO:0019901 protein kinase binding	3.63E-3
GO:0035257 nuclear hormone receptor binding	4.92E-3
GO:0070003 threonine-type peptidase activity	8.83E-3
** (b)**	
GO:0003690 double-stranded DNA binding	8.11E-8
GO:0032559 adenyl ribonucleotide binding	1.54E-5
GO:0004672 protein kinase activity	2.50E-5
GO:0010843 promoter binding	1.08E-3
GO:0019901 protein kinase binding	4.13E-3
GO:0005031 tumor necrosis factor receptor activity	4.98E-3

(a) summarizes the most significantly enriched level 5 MF (Molecular Function) GO terms for *H. sapiens* proteins involved in the stringent homology-based approach predicted host-pathogen PPI dataset using DAVID database (threshold “count >2, p-value <0.01”).

(b) summarizes the most significantly enriched level 5 MF (Molecular Function) GO terms for *H. sapiens* proteins involved in the conventional homology-based approach predicted host-pathogen PPI dataset using DAVID database (threshold “count >2, p-value <0.01”).

DAVID does not support the functional enrichment analysis of *M. tuberculosis* H37Rv proteins. Moreover, as we have found in another work [23], most of the GO annotations for *M. tuberculosis* H37Rv are not specific enough to provide effective functional enrichment analysis. Therefore the functional analysis of *M. tuberculosis* H37Rv proteins is not discussed in this work.

### Pathway enrichment analysis of proteins involved in host-pathogen PPIs

Pathway data are a primary functional source for identifying a list of proteins’ related functions. Usually for a set of proteins, if they are significantly enriched in certain pathways, it is very likely that this set of proteins play coordinated roles *in vivo*. Therefore pathway enrichment analysis is one of the most frequently used assessments on predicted host-pathogen PPIs.

For pathway enrichment analysis, we use the IntPath database [24], which is currently one of the most comprehensive integrated pathway databases. The “Identify Pathways” function in IntPath can specifically identify the pathway enrichment of an input gene list. The “Identify Pathways” function in IntPath adopts the hypergeometric test to identify the input gene list’s over-representation (enrichment) in the pathways. For each *H. sapiens* protein set (predicted by the stringent and the conventional homology-based approaches), we analyze the *H. sapiens* proteins’ pathway enrichment using the IntPath database [24], and the top 20 most significantly enriched pathways are listed in the Table 4. The enrichment analysis results summarized in the Table 4(a) and Table 4(b) provide an important evidence on which of the two approaches can predict more *H. sapiens–M. tuberculosis* H37Rv PPIs that are more relevant to *M. tuberculosis* H37Rv infection.

**Table 4:** **Pathway enrichment analysis of *H. sapiens *** **proteins involved in the predicted host-pathogen PPI dataset**

**Pathway names**	**p-value**
** (a)**	
Focal adhesion	5.85E-13
Translation factors	6.61E-12
Pathways in cancer	7.51E-12
Measles	5.21E-09
Pancreatic cancer	7.44E-09
Proteasome	8.80E-09
Antigen processing and presentation	1.68E-08
Adipogenesis	3.41E-08
Myometrial relaxation and contraction pathways	5.66E-08
MAPK signaling pathway	5.82E-08
Endocytosis	5.87E-08
Integrated cancer pathway	5.89E-08
Viral myocarditis	8.03E-08
Cell cycle	8.28E-08
Leishmaniasis	1.08E-07
T cell receptor signaling pathway	1.12E-07
Tuberculosis	2.76E-07
Spliceosome	7.79E-07
Renal cell carcinoma	7.82E-07
Amoebiasis	8.28E-07
(b)	
Hepatitis C	2.03E-14
Pathways in cancer	2.52E-13
Endocytosis	3.20E-13
MAPK signaling pathway	5.66E-13
Neurotrophin signaling pathway	4.67E-12
v Cell cycle	1.78E-11
Shigellosis	4.18E-11
T cell receptor signaling pathway	3.21E-10
Senescence and autophagy	7.20E-10
NOD-like receptor signaling pathway	9.06E-10
Prostate cancer	1.35E-09
EBV LMP1 signaling	4.64E-09
RIG-I-like receptor signaling pathway	4.74E-09
Acute myeloid leukemia	2.42E-08
Osteoclast differentiation	3.37E-08
Apoptosis	3.86E-08
Chagas disease (American trypanosomiasis)	9.86E-08
Pancreatic cancer	1.03E-07
Proteasome	1.14E-07
DNA damage response	1.25E-07

(a) summarizes the 20 most significantly enriched pathways for *H. sapiens* proteins involved in the host-pathogen PPI dataset predicted by our stringent homology-based approach.

(b) summarizes the 20 most significantly enriched pathways for *H. sapiens* proteins involved in the host-pathogen PPI dataset predicted by the conventional homology-based approach.

Besides comparing the quality of the two host-pathogen PPI datasets predicted by the two approaches based on pathway enrichment, we also analyze the pathway enrichments for the *M. tuberculosis* H37Rv proteins. This is the first-ever pathway enrichment analysis on pathogen proteins in the predicted host-pathogen PPIs. It is enabled by IntPath [24], which supports pathway analysis for this important pathogen. The pathway analysis on the *M. tuberculosis* H37Rv proteins are not used to assess the performance of the two homology-based approaches—this is the first work to analyze the pathway enrichment of the pathogen proteins, so we have no base line to compare with. The results of pathway enrichment analysis on the *M. tuberculosis* H37Rv proteins involved in *H. sapiens–M. tuberculosis* H37Rv PPIs predicted by the stringent homology-based approach are listed in Table 5.

**Table 5:** **Pathway enrichment analysis of *M. tuberculosis *** **H37Rv proteins involved in the predicted host-pathogen PPI dataset**

**Pathway names**	**p-value**
Metabolic pathways	6.81E-39
tRNA charging pathway	1.46E-18
Biosynthesis of secondary metabolites	1.54E-17
Pyrimidine metabolism	6.72E-10
Purine metabolism	2.25E-09
Aminoacyl-tRNA biosynthesis	6.47E-09
Alanine, aspartate and glutamate metabolism	3.09E-07
Superpathway of histidine, purine, and pyrimidine biosynthesis	3.25E-07
Superpathway of chorismate	1.14E-06
Arginine biosynthesis	1.39E-06
Superpathway of citrulline metabolism	2.13E-06
Tetrapyrrole biosynthesis I	2.13E-06
Tryptophan biosynthesis	2.13E-06
Phenylalanine, tyrosine and tryptophan biosynthesis	2.22E-06
Superpathway of cytosolic glycolysis, pyruvate dehydrogenase and TCA cycle	1.72E-05
Glyceraldehyde 3-phosphate degradation	3.47E-05
Gluconeogenesis I	3.92E-05
Pyrimidine ribonucleotides de novo biosynthesis	3.92E-05
Nucleotide excision repair	3.98E-05
Glycine, serine and threonine metabolism	4.53E-05

This table summarizes the 15 most significantly enriched pathways for *M. tuberculosis* H37Rv proteins involved in the predicted host-pathogen PPI dataset.

### Analysis of sequence properties of proteins involved in host-pathogen PPIs

The analysis of primary protein sequence properties considers protein sequence length, number of domains, degrees of domains on proteins, length of domains on proteins, hydrophobicity, electron charge, etc. The protein sequence properties directly reflect differences between the proteins involved in inter-species host-pathogen PPIN and intra-species PPIN. We analyze the sequence properties of both *M. tuberculosis* H37Rv and *H. sapiens* involved in the predicted host-pathogen PPIs, and compare them with other proteins in their own intra-species PPIN.

The annotation of both *M. tuberculosis* H37Rv and *H. sapiens* protein domains is accomplished using HMMER-V3.0 [25]. The domain profiles used in the protein domain annotation are from Pfam-A [26]. The threshold for the domain annotation is E-value(iE-value) ≤*E*-20 and accuracy ≥0.9. For each domain annotated on each protein, we retrieve the sequences of the domains on every protein for the following analyses.

Hydrophobicity of the proteins and domains are assessed based on the Kyte-Doolittle hydrophobicity scale. Kyte-Doolittle is a widely applied scale for delineating hydrophobic character of a protein. Regions with values above 0 are hydrophobic. We scan the sequences of the proteins and domains and calculate the average hydrophobicity scale of each protein and each domain (sum the hydrophobicity scale of each amino acid and then divide by the length of the protein/domain).

For the domain degree analysis, we obtain the DDI(Domain-Domain Interaction) data from the DOMINE database. DDIs “inferred from PDB entries” and “high confidence predictions” in the DOMINE database are considered in this study, while “medium confidence predictions” and “low confidence predictions” are discarded. For each domain, we count the number of interaction partners in the DOMINE database (only “inferred from PDB entries” and “high confidence predictions”) as the degree of that domain.

The protein/domain net charge is calculated in the following ways: only three amino acids (Arginine, Histidine, Lysine) are positively charged (assigned value +1), two amino acids (Aspartic Acid, Glutamic Acid) are negatively charged (assigned value -1), the rest amino acid are neutral (assigned value 0). The average charge of each protein/domain is calculated by scanning the protein/domain sequence and taking the average value of each protein/domain (sum the charge value divide by the length of the protein/domain).

We analyze the above protein sequence properties and summarize the results in Table 6. We conduct a similar analysis on the domains, and the results are shown in Table 7.

**Table 6:** Protein sequence properties analysis result

**Organism**	***H. sapiens *** **proteins**		***M. tuberculosis *** **proteins**	
**PPIN**	**Hum-Mtb**	**Hum-Hum**	**Hum-Mtb**	**Mtb-Mtb**
Average length	769.3	623.0	486.0	328.7
P-value	1.33E-7		7.36E-17	
Average hydrophobicity	-0.453	-0.413	-0.034	-0.027
P-value	2.39E-3		0.700	
Average charge	0.058	0.065	0.068	0.079
P-value	9.07E-4		7.31E-7	
Average No. of domains	1.39	1.31	1.55	1.25
P-value	2.65E-2		2.82E-6	
Average domain degrees	10.56	10.19	5.54	3.16
P-value	0.756		5.94E-4	

This table summarizes our analysis of protein sequence properties for *H. sapiens* and *M. tuberculosis* H37Rv proteins involved in the predicted host-pathogen PPI dataset compared with proteins involved in intra-species PPIN.

Abbreviations: Hum-Mtb: in predicted *H. sapiens–M. tuberculosis* H37Rv PPIN. Hum-Hum: in *H. sapiens * intra-species PPIN. Mtb-Mtb: in *M. tuberculosis* intra-species PPIN.

**Table 7:** Domain sequence properties analysis result

**Organism**	***H. sapiens* proteins**		***M. tuberculosis* proteins**	
**PPIN**	**Hum-Mtb**	**Hum-Hum**	**Hum-Mtb**	**Mtb-Mtb**
Average length	205.0	188.4	210.0	187.2
P-value	0.863		2.04E-2	
Average hydrophobicity	-0.355	-0.293	-0.033	0.037
P-value	2.15E-2		7.90E-4	
Average charge	0.055	0.059	0.069	0.076
P-value	4.19E-2		9.93E-3	
Average degrees	11.66	11.62	4.42	4.47
P-value	0.97		0.89	

This table summarizes our analysis of domain sequence properties for *H. sapiens* and *M. tuberculosis* H37Rv proteins involved in the predicted host-pathogen PPI dataset, compared with proteins involved in intra-species PPIN.

Abbreviations: Hum-Mtb: in predicted *H. sapiens–M. tuberculosis* H37Rv PPIN. Hum-Hum: in *H. sapiens * intra-species PPIN. Mtb-Mtb: in *M. tuberculosis* intra-species PPIN.

### Analysis of intra-species PPIN topological properties in host-pathogen PPIs

Intra-species PPIN topological properties examined and reported by Calderwood *et al.*[27] and then repeatedly confirmed by others [5]. In this work, we also conduct a similar study on the targeted *H. sapiens* proteins by examining the number of interaction partners in the intra-species PPIN. Previous analyses are mainly constrained on the *H. sapiens* proteins as the *H. sapiens* PPIN are ready to use, while most of the pathogen’s intra-species PPIs are not available. Due to Zhou *et al*’s [23] work on *M. tuberculosis* H37Rv intra-species PPIN, a high quality *M. tuberculosis* H37Rv PPI dataset is now available. Therefore this work is among the few studies that examines the intra-species PPIN topological properties of the pathogen proteins involved in host-pathogen PPIs.

We mainly consider three important topological properties, Degree (the number of interaction partners in the intra-species PPIN), Betweenness Centrality (a measure of a node’s centrality in a network, equal to the number of shortest paths from all vertices to all others that pass through that node in the intra-species PPIN), Shortest Path Length (average number of steps along the shortest paths for all possible pairs of network nodes, it measures the efficiency of information transport on a network). All these topological properties are calculated using Cytoscape’s [14] Analyze Network Plugin.

In this work, *H. sapiens* intra-species PPIs are collected mainly from three databases, MINT [15], BioGRID [16], and IntAct [17]. *M. tuberculosis* H37Rv PPIs are collected from STRING (with score above 770) [28] and the B2H PPI dataset (four small subsets of reliable PPIs) [23].

The results are shown in Table 8.

**Table 8:** Topological properties analysis result

**Organism**	***H. sapiens* proteins**		***M. tuberculosis* proteins**	
**PPIN**	**Hum-Mtb**	**Hum-Hum**	**Hum-Mtb**	**Mtb-Mtb**
Average degree	26.69	12.56	25.67	16.16
P-value	2.18E-11		7.34E-9	
Average betweeness centrality	6.33E-4	8.23E-4	8.36E-3	1.63E-2
P-value	0.439		0.132	
Average shortest path length	3.33	3.57	4.73	4.77
P-value	1.33E-30		0.65	

This table summarizes our analysis of intra-species PPIN topological properties for *H. sapiens* and *M. tuberculosis* H37Rv proteins involved in the predicted host-pathogen PPI dataset, compared with proteins involved in intra-species PPIN.

Abbreviations: Hum-Mtb: in predicted *H. sapiens–M. tuberculosis* H37Rv PPIN. Hum-Hum: in *H. sapiens* intra-species PPIN. Mtb-Mtb: in *M. tuberculosis* intra-species PPIN.

### Software packages and datasets

The software packages and database tools used in this study are: 

• IntPath [24]

• BBH-LS [18]

• Cytoscape [14]

• HMMER-V3.0 [25]

• DAVID [22]

The datasets used in this study are: 

• *M. tuberculosis* H37Rv PPI dataset consisting of four reliable subsets of the B2H PPI dataset and STRING PPI dataset (threshold at 770) [23].

• *H. sapiens* PPI dataset collected from MINT [15], BioGRID [16], and IntAct [17], date of download is November 10, 2011.

• Host-pathogen PPI data from PATRIC [13], date of download is April 3, 2012.

• 10 bacteria gene feature files, and whole genome fasta files are from PATRIC [13], date of download is April 3rd, 2012.

• DDI data from DOMINE database V2.0 [29].

• Pfam-A Domain profiles. [26]

• *H. sapiens*–HIV-1 PPI dataset downloaded from “HIV-1, human protein interaction database at NCBI” [30].

## Results

### Prediction of host-pathogen PPI network

For our stringent homology-based approach, the most abundant template eukaryote-prokaryote inter-species PPIs are between human and 10 major bacteria species (7120 PPIs). Therefore when predicting the *H. sapiens–M. tuberculosis* H37Rv PPIs we only need to identify the prokaryotic homologs between template and targeted species in this situation. We identify, using BBH-LS (strength threshold *β*≥0.01), the homologs between *M.tuberculosis* H37Rv and the 10 bacteria from the PATRIC database. Here in this work we use the “consensus score” (the number of supporting template PPIs) as one of the parameters to evaluate how likely a predicted PPI is real, compared to the other predicted PPIs. For example, if there are 3 template human-bacteria PPIs transferring to the same *H. sapiens–M. tuberculosis* H37Rv PPI, then the PPI’s consensus score is “3”. A total of 1005 *H. sapiens–M. tuberculosis* H37Rv PPIs are transferred from 7120 eukaryote-prokaryote (human-pathogen) PPIs. A visualization of the *H. sapiens-M. tuberculosis* H37Rv PPIN are shown in Figure 2. The blue dots are *M. tuberculosis* H37Rv proteins, while the orange dots are *H. sapiens* proteins. The “thickness” of an edge corresponds to the “consensus score” of each predicted H. sapiens-M. tuberculosis H37Rv PPI. The predicted *H. sapiens-M. tuberculosis* H37Rv PPI dataset can be found in the Additional file 1.

For the conventional homology-based approach we obtain 73251 template PPIs from MINT, BioGRID and IntAct. We identify the homologs between human and *M.tuberculosis* to transfer PPIs in the prediction. Using BBH-LS (strength threshold *β*≥0.01), we identify 355 homologs between *M.tuberculosis* H37Rv and *H. sapiens*. Using these 355 homologs, we predict 326 *H. sapiens–M. tuberculosis* H37Rv PPIs from the 73251 eukaryote-eukaryote (human-human) intra-species PPIs.

The number of templates we start from and the number of predicted PPIs are surprisingly different between the stringent homology-based approach and the conventional homology-based approach. Using the same system and threshold in identifying homologs and then transferring a template PPI to predict a target host-pathogen PPI, in the stringent homology-based approach, 1005 inter-species PPIs are predicted from 7120 template PPIs; while in the conventional homology-based approach, only 326 inter-species PPIs are predicted from 73251 template PPIs. This result shows that our stringent homology-based approach are more efficient in using the template PPIs than the conventional homology-based approach in predicting prokaryote-eukaryote inter-species PPIs. This highlights the huge potential of our stringent homology-based approach in applying to many host-pathogen systems.

### Cellular compartment distribution of *H. sapiens* proteins targeted by predicted host-pathogen PPIs.

The cellular compartment of the *H. sapiens* proteins targeted by the predicted host-pathogen PPIs can provide important clues about the quality of the *H. sapiens-M. tuberculosis* H37Rv PPIs predicted. If the targeted *H. sapiens* proteins are mostly located in cellular compartments having a close relationship with pathogen infection or known interactions with host cells, then we can be more certain about the quality of our results.

We identify the informative CC terms of the *H. sapiens* proteins. Then we calculate the number and percentage of proteins in the datasets that have been annotated with each of the informative CC terms (Additional file 3). Then we plot the top 10 most frequently located informative CC terms for the targeted *H. sapiens* proteins by the stringent and the conventional homology-based approach in Figure 3 and Figure 4. We also summarize the statistics into Table 1.

Many of the host-pathogen PPIs predicted by the stringent homology-based approach target *H. sapiens* proteins locate in very relevant cellular compartments. This corresponds to the pathogen’s infection and invasion of host cells. Among the top ten most frequent cellular compartment (GO) terms, four of them are closely relevant to the *M. tuberculosis* H37Rv infection. Those four terms are: extracellular space (GO:0005615), transcription factor complex (GO:0005667), proteasome complex (GO:0000502), external side of plasma membrane (GO:0009897).

*H. sapiens* proteins at extracellular space (GO:0005615) and extracellular space membrane (GO:0009897) have a much higher chance to interact with the pathogen *M. tuberculosis* H37Rv, because invasive bacteria pathogens are more likely to interact with the receptors, outer membrane proteins located on these two cellular compartments. The CC term, transcription factor complex (GO:0005667), is also relevant to *M. tuberculosis* infection, as *M. tuberculosis* has close interplay with *H. sapiens* cells on the transcription process.

For example, *M. tuberculosis* infection of human macrophages blocks several responses to IFN- *γ*. The inhibitory effect of *M. tuberculosis* is directed at the transcription of IFN- *γ*-responsive genes [31]. There is a marked decrease in IFN- *γ* induced association of STAT1 with the transcriptional coactivators CREB-binding protein and p300 in *M. tuberculosis*-infected macrophages, indicating that *M. tuberculosis* directly or indirectly disrupts this protein-protein interaction that is essential for transcriptional responses to IFN- *γ*[31]. Several studies show that infection with *M. tuberculosis* increases the replication of HIV in mononuclear cells [32]. It turns out that *M. tuberculosis* and its purified protein derivative induced HIV LTR [32]. And the effect of *M. tuberculosis* and its purified protein derivative on HIV replication in monocytes is primarily one of transcriptional activation [32].

The CC term proteasome complex (GO:0000502), is also strongly related to *M. tuberculosis* infection. It is found that the interaction between the mycobacterial phagosome and the endoplasmic reticulum lead to proteasome degradation and MHC class I presentation of *M. tuberculosis* antigens. Thus, the results shown in Table 1(a) strongly supports the validity of our prediction results using the stringent homology-based prediction approach.

In contrast, there are three relevant CC terms out of the top ten most frequent cellular compartments where the conventional homology-based approach predicted host-pathogen PPIs targeted *H. sapiens* proteins locate at. These terms are: transcription factor complex (GO:0005667), mitochondrial matrix (GO:0005759), mitochondrial outer membrane (GO:0005741); see Table 1(b).

*M. tuberculosis* H37Rv infection has a close relationship with mitochondria activities and function and was shown to induce quantitatively distinct changes in the mitochondrial proteome [33]; therefore mitochondrial matrix (GO:0005759) and mitochondrial outer membrane (GO:0005741) are relevant to *M. tuberculosis* H37Rv infection. It is found that mitochondria in *M. tuberculosis* H37Rv-infected cells displayed robust activity with increased membrane potential and ATP synthesis [33]. Ultrastructural changes in the mitochondria and mitochondrial clustering are also observed in the *M. tuberculosis* H37Rv infected cells [33]. The augmentation of mitochondrial activity by *M. tuberculosis* H37Rv enables manipulation of host cellular mechanisms to inhibit apoptosis and ensure fortification against anti-microbial pathways [33].

From the results we can tell that, the stringent homology-based approach has a better performance in predicting *H. sapiens-M. tuberculosis* H37Rv PPIs comparing with that of the conventional homology-based approach.

### Disease-related enrichment analysis of proteins involved in host-pathogen PPIs

The disease-related enrichment analysis results of *H. sapiens* proteins in *H. sapiens–M. tuberculosis* H37Rv PPIs predicted by the stringent homology-based approach show significant enrichment in all the gene lists, as summarized in Table 2(a). The significant enrichment of *H. sapiens* proteins involved in host-pathogen PPIs in “early, late, consistent changers” gene lists [19] and also in “Macrophages and dendritic differentially expressed genes” [20] is further evidence that *H. sapiens-M. tuberculosis* H37Rv PPIs predicted by our stringent homology-based approach are valid and very relevant to the infection process of *M. tuberculosis* H37Rv. This result has obvious biological basis.

In contrast, the results from the conventional homology-based approach show much less significant enrichment than the results from the stringent homology-based approach; see Table 2(b). This comparison clearly shows that our stringent homology-based approach has much better performance than the conventional homology-based approach.

### Functional enrichment analysis of proteins involved in host-pathogen PPIs

Functional enrichment analysis points out the possible functional relevance of *H. sapiens* proteins involved in the *H. sapiens-M. tuberculosis* H37Rv PPIN predicted by both the stringent and the conventional homology-based approaches. The representative result—the most significantly enriched level 5 MF GO terms—are listed in Table 3. From the enrichment analysis results of the *H. sapiens* proteins targeted by the stringent homology-based approach predicted PPIs, shown in Table 3(a), five out of six significantly enriched terms are strongly *M. tuberculosis* H37Rv infection related functional categories, namely “GO:0051015 actin filament binding”, “GO:0010843 promoter binding”, “GO:0003713 transcription coactivator activity”, “GO:0019901 protein kinase binding”, “GO:0035257 nuclear hormone receptor binding”.

During vesicular fusion, the movement of endosomes and lysosomes are guided by the actin molecules associated with them. The fusion of endosomes with lysosomes is seriously affected by the disruption of actin filaments. And it has been reported that host cell’s actin filament network are found to be interfered by pathogenic species of mycobateria [34-36]. A more recent study shows that *M. tuberculosis* affects actin polymerisation [37]. Therefore the functional enrichment analysis strongly supports the validity of the prediction results from our stringent homology-based approach, as the most enriched MF term shown in Table 3(a) is “actin filament binding” (GO:0051015).

The significant enrichment of the terms “promoter binding (GO:0010843)”, “transcription coactivator activity (GO:0003713)” are closely related to *M. tuberculosis* infection, which also supports the validity of the prediction results by our stringent homology-based approach. As discussed above, *M. tuberculosis* infection of human macrophages has inhibitory effect on transcription of IFN- *γ*-responsive genes [31]. It directly or indirectly influences transcriptional responses to IFN- *γ*[31]. And *M. tuberculosis* increases the replication of HIV in mononuclear cells [32]. The effect of *M. tuberculosis* and its purified protein derivative on HIV replication in monocytes is primarily one of transcriptional activation [32].

Bacterial pathogens have many ways to target one of the most ubiquitous signaling mechanisms of all eukaryotic host: phosphorylation by protein kinases [38]. MAPKs are evolutionarily conserved kinases that are important in cellular signal transduction [2]. There are three main families of MAPKs: (i) the c-Jun N-terminal kinases; (ii) the extracellular signal-related kinases; (iii) the p38 MAPK [2]. Many bacterial pathogens (including *M. tuberculosis*) modify MAPK signalling to promote their survival in the host cells [2]. By usurping p38 to interfere with CD1 surface expression, mycobacteria disrupt MAPK signaling pathways which play a crucial role in immune modulation [38,39]. And p38 is predicted to be targeted by *M. tuberculosis* H37Rv by our stringent homology-based approach. Therefore it is very reasonable and meaningful for the targeted host proteins to have significant functional enrichment in the term “GO:0019901 protein kinase binding”. *M. tuberculosis* and its components are strong inducers of cytokines, such as tumour necrosis factor-alpha (TNF- *α*) and IL-1 *β*[40,41].

Many nuclear hormone receptors are shown to play a role in the repression of inflammatory mediators and they are also capable of modulating innate immunity in a positive manner [42]. Liu *et al.*[43] demonstrated, through the upregulation of VDR and vitamin D-1-hydroxylase genes, that TLRs adopt VDR antimicrobial activity in response to *M. tuberculosis* infection [42]. Therefore the evidence is clear that, through positive and negative regulatory mechanisms, nuclear hormone receptors regulate innate immune responses to bacteria infections [42]. This makes sense as this functional category of *H. sapiens* proteins are likely to be targeted by *M. tuberculosis* H37Rv proteins during infection.

In contrast, in the enrichment analysis results of *H. sapiens* proteins targeted by the conventional homologybased approach predicted PPIs, show in Table 3(b), only four out of six significantly enriched terms are strongly *M. tuberculosis* H37Rv infection related functional categories, including “GO:0004672 protein kinase activity”, “GO:0010843 promoter binding”, “GO:0005031 tumor necrosis factor receptor activity”, “GO:0019901 protein kinase binding”.

This functional enrichment analysis shows that our stringent homology-based approach is more accurate, and has merits in identifying possible *H. sapiens* proteins that are involved in *H. sapiens–M. tuberculosis* H37Rv PPIs.

### Pathway enrichment analysis of proteins involved in host-pathogen PPIs

Pathway enrichment analysis of the proteins involved in host-pathogen PPIN can tell a lot about the functional relevance of (both the host and pathogen) proteins involved in the host-pathogen PPIN. Therefore, pathway enrichment analysis has been used frequently in assessing host-pathogen PPI prediction results. The assessment stems from the basis that the host proteins involved in host-pathogen interactions should be a set of proteins that have functional correlation to pathways relevant to the pathogen’s infection. So we also conduct pathway enrichment analysis to assess the quality of our prediction results and the performance of both the stringent and the conventional homology-based prediction approaches.

For *H. sapiens* proteins involved in the *H. sapiens-M. tuberculosis* H37Rv PPIN predicted by the stringent homology-based approach, they are mostly enriched in pathways that are closely relevant to *M. tuberculosis* infection. Among the top 20 most significantly enriched pathways, 13 are closely relevant to *M. tuberculosis* infection; see Table 4(a). For example, “Amoebiasis”, “Measles”, “Tuberculosis”, “Antigen processing and presentation”, “Viral myocarditis”, “Leishmaniasis”, and “T cell receptor signaling pathway” are strongly infectious disease related and immune response related pathways which are obviously very relevant to *M. tuberculosis* infection. Moreover, our stringent homology-based approach predicted *H. sapiens* protein targets that are significantly enriched in the “Tuberculosis” pathway, which is a strong evidence supporting our prediction approach. “Focal adhesion”, “Spliceosome”, “Proteasome”, “MAPK signaling pathway”, and “Endocytosis” are essential pathways closely interconnected to the “Tuberculosis” pathway. These essential pathways play crucial roles in the *M. tuberculosis* infection process and in the immune response to the infection.

The “Focal adhesion” pathway is closely interconnected to the *M. tuberculosis* infection process. In many bacterial pathogens, protein tyrosine phosphatases (PTPases) have been demonstrated to be essential for dephosphorylating host focal adhesion proteins and focal adhesion kinase. This dephosphorylation leads to destabilization of focal adhesions which are involved in the internalization of bacterial pathogens by eukaryotic cells [44,45]. There are two functional PTPases in *M. tuberculosis*[46]. A very interesting fact is that the *M. tuberculosis* genome lacks tyrosine kinases; so the existence of these two secretory tyrosine phosphatases (PTPases) shows that they are very likely involved in the dephosphorylation of host proteins. A study shows that, when the mptpB gene is deleted from *M. tuberculosis*, the mutant strain is attenuated in the lung and spleen of infected animals [47]. Therefore the “Focal adhesion” pathway is a very important target for *M. tuberculosis* infection of host. The significant enrichment of this pathway strongly supports the validity of the prediction results of our stringent homology-based approach, as shown in Table 4(a).

The invasion of *M. tuberculosis* to the host cell is closely facilitated by endocytosis, which is one of early steps for the pathogen to interact with proteins inside the host cell. Proteasome is also strongly related to *M. tuberculosis* infection. It is found that the interaction between the mycobacterial phagosome and the endoplasmic reticulum leads to proteasome degradation and MHC class I presentation of *M. tuberculosis* antigens.

MAPKs are evolutionarily conserved kinases that are important in cellular signal transduction [2]. Many bacterial pathogens (including *M. tuberculosis*) modify MAPK signalling to promote their survival in the host cells [2].

From the biological aspect, the *H. sapiens* proteins involved in the *H. sapiens-M. tuberculosis* H37Rv PPIs (predicted by the stringent homology-based approach) are likely to be involved in the above enriched pathways. This pathway enrichment analysis suggests that our stringent homology-based prediction accurately identifies *H. sapiens* proteins that are likely to be targeted by *M. tuberculosis* H37Rv.

In contrast, the pathway enrichment analysis of *H. sapiens* proteins involved in the *H. sapiens-M. tuberculosis* H37Rv PPIN predicted by the conventional homology-based approach shows that the conventional homology-based approach does not have the same good performance as the stringent homology-based approach. Among the top 20 most significantly enriched pathways, only 9 are closely relevant to *M. tuberculosis* infection; see Table 4(b). For example, “Hepatitis C”, “Shigellosis”, “T cell receptor signaling pathway”, “EBV LMP1 signaling”, and “Chagas disease (American trypanosomiasis)” are infectious disease related and immune response related pathways relevant to *M. tuberculosis* infection. “Endocytosis”, “MAPK signaling pathway”, “Apoptosis”, and “Proteasome” are essential pathways also considered as related pathways. This comparative analysis shows both homology-based approaches can predict the *H. sapiens-M. tuberculosis* H37Rv PPIN and pathway enrichment analysis supports both prediction results. However, apparently the stringent homology-based approach has much better performance than that of the conventional homology-based approach.

**Figure 5:**
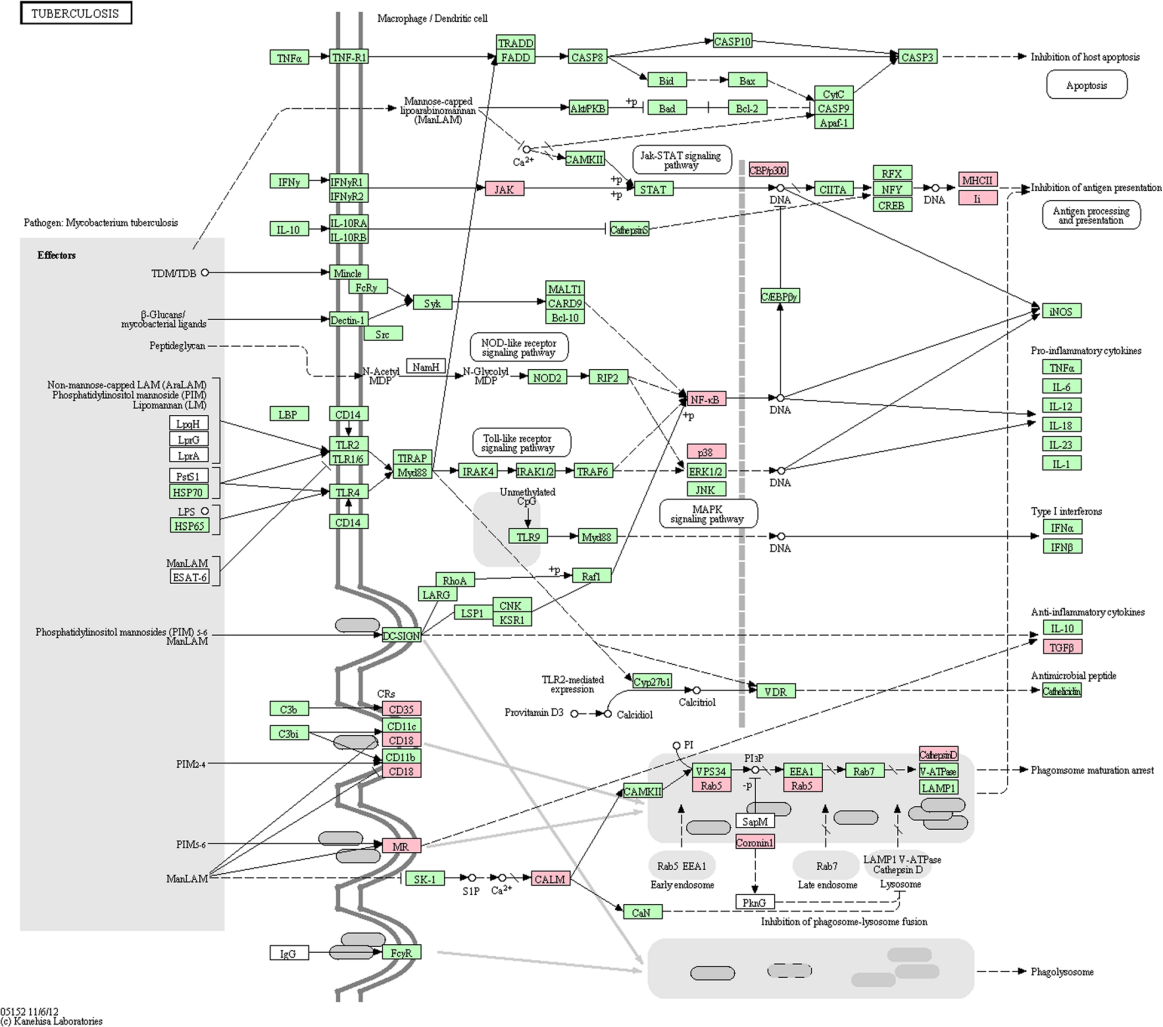
**Visualization of the KEGG “Tuberculosis” pathway with *H. sapiens *proteins recovered by our predicted *H. sapiens-M. tuberculosis *H37Rv PPI network.** The pink squares are *H. sapiens * proteins targeted in our predicted *H. sapiens-M. tuberculosis* H37Rv PPIN that are in the KEGG “Tuberculosis” pathway map. The green squares are *H. sapiens* proteins in the “Tuberculosis” pathway, but not recovered in our prediction.

Among the most significantly enriched pathways, our stringent homology-based approach recovers the “Tuberculosis” pathway. We use the KEGG pathway map [48] to visualize the *H. sapiens* proteins that are targeted in our prediction results (in pink color) and all rest of the proteins participating in the pathway (in green color). The pathway map is shown in Figure 5. For those *H. sapiens* proteins in this pathway that are targeted by the *H. sapiens-M. tuberculosis* H37Rv PPIs (predicted by the stringent homology-based approach), we summarized the PPIs into Table 9.

**Table 9:** Human proteins in Tuberculosis pathway that are targeted by the predicted host-pathogen PPIs

***H. Sapiens *** **protein**	***M. Tuberculosis *** **protein**	**Consensus score**	**Cellular compartment**	**Molecular function**
CTSD	Rv2987c	3	GO:0005737 cytoplasm	GO:0016787 hydrolase activity
NFKB1	Rv0155	3	GO:0005737 cytoplasm	GO:0005515 protein binding
CR1	Rv1589	3	GO:0044459 plasma membrane part	GO:0005515 protein binding
ITGB2	Rv1133c	3	GO:0005737 cytoplasm	GO:0005515 protein binding
CD74	Rv0685	1	GO:0005737 cytoplasm	GO:0005515 protein binding
RAB5A	Rv1020	2	GO:0005737 cytoplasm	GO:0005515 protein binding
RAB5C	Rv1122	3	GO:0005737 cytoplasm	GO:0005515 protein binding
JAK1	Rv1340	3	GO:0005737 cytoplasm	GO:0005515 protein binding
TGFB1	Rv1384	3	GO:0005737 cytoplasm	GO:0005515 protein binding
CORO1A	Rv0685	1	GO:0005737 cytoplasm	GO:0005515 protein binding

This table summarizes the human proteins that are targeted by the predicted host-pathogen PPIs.

It is known that *M. tuberculosis* virulence factor inhibits the accumulation of syntaxin 6 and Cathepsin D(CTSD) by latex bead phagosomes [49]. It is likely that this important host protein CTSD might also be targeted by *M. tuberculosis* proteins to facilitate the successful *M. tuberculosis* infection to human. The survival of *M. tuberculosis* will be significantly decreased if Nuclear Factor-Kappa B (NFKB1) activation are inhibited [50]. Therefore, it is plausible that *M. tuberculosis* interferes NF *κ*B activation through the binding of Rv0155 and NFKB1. Complement Receptor 1 (CR1) has been implicated in playing a role in *M. tuberculosis* adherence [51]. This supports the plausibility that CR1 might interact with Rv1589 during the infection of *M. tuberculosis*. This PPI may be very important during *M. tuberculosis* adherence to host, it may also be related with *M. tuberculosis* resistance to host immune response and clearance. The induction of transcripts encoding CD18 (ITGB2) on D21 in a *M. tuberculosis*-infected lung [52] may partially supports our hypothesis that Rv1133c interacts with ITGB2 during the *M. tuberculosis* infection to the lung. A recent work shows that *M. tuberculosis* ppiA (Rv0009) interacts with CD74 and meanwhile Rv0009 also interacts with Rv0685 [53]. Therefore it is plausible that CD74 might interact with Rv0685. Phagosomes containing live *M. tuberculosis* acquire RAB5A involved in transport between late endosomes and lysosome [54]. This creates an opportunity for RAB5A to interact with *M. tuberculosis*, therefore it is very plausible that RAB5A may be targeted by Rv1020. *M. tuberculosis* has been shown to induce IL-10 production and suppress the production of IL-12 and TNF- *α*. IL-6 and IL-12 induce the expression of Rab5c and Rab7. Because Rab5c has been induced and highly expressed, there is a more abundance of Rab5c that might be able to interact with *M. tuberculosis*. Activation of JAK2/STAT1- *α*-dependent signaling events has been observed during *M. tuberculosis* induced macrophage apoptosis and activation of JAK1/STAT1- *α* is essential for the induction of the intracellular events occurring after *M. tuberculosis* infection [55]. It is found that local pulmonary immunotherapy with siRNA targeting TGFB1 enhances antimicrobial capacity in *M. tuberculosis* infected mice [56]. Therefore, it is very likely that the interaction between TGFB1 and Rv1384 play an important role in the *M. tuberculosis* infection. It is shown that CORO1A inhibits autophagosome formation around *M. tuberculosis*-containing phagosomes and assists mycobacterial survival in macrophages [57]. Therefore it is a very interesting discovery that CORO1A might potentially interact with Rv0685, and this interaction may partially contribute to *M. tuberculosis* survival. Therefore our *H. sapiens–M. tuberculosis* H37Rv PPIs are very plausible and supported by evidence above.

Some cancer-related pathways are also present in the list of most enriched pathways. The presence of cancer pathways may or may not be regarded as artifacts of the pathway analysis. On one hand, cancers share lots of similarity with pathogen infection, including evading immune response, inducing apoptosis, metastasis and invading the cells, etc. Therefore, many essential pathways that are highly interconnected to *M. tuberculosis* infection are also closely related to cancer pathways. Those essential pathways are “Apoptosis”, “MAPK signaling pathway”, “Jak-STAT signaling pathway”, “Focal adhesion”, etc. On the other hand, presence of cancer pathways in the highly enriched pathways is also caused by the overlap of many “core” proteins, which mostly are the house keeping genes of *H. sapiens* cells.

*M. tuberculosis* H37Rv proteins involved in the stringent homology-based approach predicted *H. sapiens-M. tuberculosis* H37Rv PPIN are mostly enriched in pathways that are related to “general metabolism”, “amino acid metabolism”, “ribonucleotides metabolism”, etc.; see Table 5. This makes sense as the pathogen infecting the human host undergoes rigorous metabolism in order to multiply and further infects the host.

Therefore the prediction results from our stringent homology-based approach can serve as a reliable reference of PPIs between *H. sapiens* and *M. tuberculosis* H37Rv.

This analysis result is in accord with the above cellular compartment distribution, disease gene list, pathway enrichment and functional category enrichment analysis results. All the results support the validity of the *H. sapiens–M. tuberculosis* H37Rv PPIs predicted by our stringent homology-based approach. Furthermore, all the analysis results above suggest our stringent homology-based approach has better performance than the conventional homology-based approach in predicting host-pathogen PPIs.

### Analysis of protein sequence properties of proteins involved in host-pathogen PPIs

The analysis of the sequence properties of proteins involved in host-pathogen PPI network reveals many interesting properties that have not been reported before. In the analysis we compare several important features of both *H. sapiens* and *M. tuberculosis* H37Rv proteins/domains in the predicted *H. sapiens–M. tuberculosis* H37Rv PPIN and their own intra-species PPIN. Table 6 provides summary results from the analysis of *H. sapiens* and *M. tuberculosis* H37Rv proteins.

It is very obvious that in the predicted *H. sapiens–M. tuberculosis* H37Rv PPIN, *H. sapiens* proteins tend to have longer primary sequence, tend to have more domains, tend to have lower charge and tend to be more hydrophilic than those proteins in the intra-species *H. sapiens* PPIN. For *M. tuberculosis* H37Rv proteins, similar properties are also exhibited; for example, *M. tuberculosis* H37Rv proteins in the predicted *H. sapiens–M. tuberculosis* H37Rv PPIN tend to have longer primary sequences, tend to have more domains, tend to have lower charge and tend to be more hydrophilic than those proteins in the intra-species *M. tuberculosis* H37Rv PPIN. When we zoom in from the protein level to the domain level, we find the domains also exhibit similar properties as the proteins; see Table 7. The most significant properties for the domains in inter-species host-pathogen PPIN are that they tend to be more hydrophilic and tend to have lower charge than counterparts in the intra-species PPIN (both in *H. sapiens* and *M. tuberculosis* H37Rv proteins).

The discoveries found by analyzing sequence properties may be helpful in illuminating the basic mechanisms of how the host and pathogen proteins interact with each other, and may be useful in assessing the predicted host-pathogen PPIN.

### Analysis of intra-species PPIN topological properties in host-pathogen PPIs

The results from the analysis of intra-species PPIN topological properties for *H. sapiens* and *M. tuberculosis* H37Rv proteins involved in the predicted host-pathogen PPI dataset in comparison with proteins involved in intra-species PPIN are summarized in Table 8.

From the intra-species PPIN topological properties of *H. sapiens* proteins involved in the predicted and gold standard host-pathogen PPINs, we conclude that *H. sapiens* proteins being targeted by pathogen proteins in the host-pathogen PPIs tend to have much higher degree than proteins in the intra-species PPIN. In other words, the host proteins being targeted by pathogens are more likely to be hubs in their own intra-species PPIN. This result further strengthens the discoveries first reported by Calderwood *et al.*[27] and is also in agreement with many studies that followed [5].

In this work we are the first to examine the intra-species PPIN topological properties of *M. tuberculosis* H37Rv proteins involved in the *H. sapiens–M. tuberculosis* H37Rv PPIN. We find that *M. tuberculosis* H37Rv proteins involved in the host-pathogen PPIN also tend to have much higher degrees than proteins in the intra-species *M. tuberculosis* H37Rv PPIN. This shows that pathogen proteins involved in the host-pathogen PPIN are also more likely to be hubs in their own intra-species PPIN.

This makes sense as pathogen proteins that interact with human proteins may also have very important functions in the pathogen’s own metabolism, and the interaction between hub pathogen proteins with host proteins may be important to switching the pathogen status from managing its own “free-living” metabolism to an “infection-oriented” metabolism.

## Discussion

### Homology-based prediction

The homology-based approach for predicting the conserved intra-species PPIs across closely related species was reported more than a decade ago [6], with the assumption that the interaction between a pair of proteins in one species is expected to be conserved in related species. It has also been widely used in predicting inter-species PPIs [7-11].

However, the limitation of the conventional homology-based approach for predicting inter-species (host-pathogen) PPIs have not been fully discussed. In particular, when applying this approach in predicting eukaryote-prokaryote PPIs, (i) the differences between eukaryotic and prokaryotic proteins and (ii) the differences between inter-species and intra-species PPI interfaces may all contribute to the limited performance of the conventional homology-based prediction approach in predicting eukaryote-prokaryote host-pathogen PPIs. Therefore, our proposed stringent homology-based prediction approach has merits in overcoming the above two limitations, and should be suitable for predicting eukaryote-prokaryote host-pathogen PPIs in many host-pathogen systems. The only limitation of our stringent homology-based approach lies in the fact that there is a limited amount of source eukaryote-prokaryote PPIs available currently. However, with the rapid advance in technology and the community’s increasing interest on host-microbe interaction studies, the eukaryote-prokaryote template PPIs will be much more abundant in the future. This should greatly facilitate the massive application of our stringent prediction approach to many host-pathogen systems in the future.

As a matter of fact, our stringent homology-based approach may not only have merits in predicting eukaryote-prokaryote PPIs, but also can be extended to many other types of inter-species PPI prediction, including eukaryote-archea PPIs, eukaryote-virus PPIs, etc. This can be especially meaningful for predicting human-virus PPIs because (i) there are large differences between human and virus proteins, (ii) human-virus PPI interfaces are also very different from intra-species PPI interfaces, and (iii) abundant template human-virus PPIs are available.

### Cancer pathways and enrichment analysis

In several host-pathogen interaction studies, when analyzing the pathway enrichment of host-pathogen PPIN targeted human proteins, cancer-related pathways also show up in the list of most enriched pathways [58]. According to our study, the presence of cancer pathways makes sense, as cancer shares many similarities with pathogen infection, including evading immune response, inducing apoptosis, metastasis and invading the cells. Therefore many essential pathways that are highly interconnected to *M. tuberculosis* infection are also closely related to cancer pathways. These essential pathways are “Apoptosis”, “MAPK signaling pathway”, “Jak-STAT signaling pathway”, “Focal adhesion”, etc. On the other hand, cancer pathways may also be an artifact because a substantial number of proteins are in the overlap between the cancer-related pathways and the essential pathways. We conduct some experiments to test this hypothesis. We group all the essential pathways that are related to *M. tuberculosis* infection, and name the collection “infection-related pathways”. The collection includes the following pathways, “Focal adhesion”, “Proteasome”, “Antigen processing and presentation”, “MAPK signaling pathway”, “Endocytosis”, “T cell receptor signaling pathway”, “Spliceosome”, “Apoptosis”, “Tuberculosis”. We also choose one large representative cancer pathway (“Pathways in cancer”). We then test the overlap of these two collections of pathways. The results of the analysis are summarized in Table 10. From the results we can see that among the 1082 proteins in “infection-related pathways” and the 326 proteins in “Pathways in cancer”, there are 169 proteins overlapping between the two datasets. The *H. sapiens–M. tuberculosis* H37Rv PPIN predicted by the stringent homology-based prediction approach involves 755 *H. sapiens* proteins. This set of 755 *H. sapiens* proteins covers 204 proteins in “infection-related pathways” and covers 29 proteins in the “Pathways in cancer”. Among these 204 and 29 proteins, 20 of them overlap with each other, this significantly demonstrates our hypothesis that the cancer-related pathways are enriched due to the substantial overlap (in protein members) with infection-related pathways (p-value ≤ 1.82E-6).

**Table 10:** **Gene content of cancer pathways and *M. tuberculosis *** **infection related pathways**

**Pathways**	**Infection related**	**Pathways in**
	**pathways**	**cancer**
Gene No.	1082	326
Overlap between pathways in cancer and infection related pathways	169	
Hum-Mtb targeted Human proteins Overlap with HP-PPI targeted human proteins	204	29
Overlap of the three datasets	20	

This table summarizes the gene content of cancer pathways and *M. tuberculosis* infection related Pathways. We choose one large representative cancer pathway—“Pathways in cancer”. The *M. tuberculosis * infection related pathways (“infection-related pathways” for short) are: “Focal adhesion, “Proteasome”, “Antigen processing and presentation”, “MAPK signaling pathway”, “Endocytosis”, “T cell receptor signaling pathway”, “Spliceosome”, “Apoptosis”, and “Tuberculosis”. Hum-Mtb: predicted *H. sapiens–M. tuberculosis* H37Rv PPIN.

### Impact and possible application of the illuminated sequence and topological properties

Among the key contributions of this work are the discoveries of sequence and topological properties of the proteins involved in the host-pathogen PPIN. Based on the analysis of our predicted host-pathogen PPINs, we see that both host and pathogen proteins involved in host-pathogen PPINs tend to have longer primary sequences, tend to have more domains, tend to have lower charge and tend to be more hydrophilic than proteins in intra-species PPINs. We also see that not only host proteins but also pathogen proteins involved in host-pathogen PPINs tend to be hubs in their own intra-species PPINs.

These important properties have big potential in application to host-pathogen interaction studies. For example, for assessing the quality of newly predicted or experimentally derived host-pathogen PPIs, we can specifically analyze the sequence and topological properties (primary protein sequences, number of domains, hydrophobicity, charge, domain degrees and intra-species PPIN degrees) of the host and pathogen proteins involved in the host-pathogen PPIs to see how likely the host-pathogen PPIN is valid. These will open more doors for the analysis and assessment of host-pathogen PPINs.

## Conclusion

In this work we have proposed a stringent homology-based approach for predicting host-pathogen PPIs. Our approach specifically overcomes the limitation of the conventional homology-based approach by taking into account two important factors: (i) differences between eukaryotic and prokaryotic proteins, and (ii) differences between intra-species and inter-species PPI interfaces.

Using this stringent homology-based approach, we have predicted 1005 *H. sapiens-M. tuberculosis* H37Rv PPIs. Pathway enrichment analysis, functional enrichment analysis, disease-related gene list enrichment analysis, etc. all support the plausibility of our prediction results and show that our stringent homology-based approach has better performance in predicting *H. sapiens–M. tuberculosis* H37Rv PPIs than the conventional homology-based approach. The *H. sapiens-M. tuberculosis* H37Rv PPI dataset predicted by our stringent homology-based approach can be used as an important reference for a variety of related studies on *H. sapiens–M. tuberculosis* H37Rv interactions, *M. tuberculosis* H37Rv infections and infectious disease prevention. However, the analyses we have performed to assess the validity of our predictions are based on strong indirect evidence. We have not been able to find large-scale experimental data that demonstrate the direct physical binding of the *H. sapiens–M. tuberculosis* H37Rv PPIs predicted here.

We have further analyzed the sequence and topological properties of both the *H. sapiens* and *M. tuberculosis* H37Rv proteins involved in *H. sapiens-M. tuberculosis* H37Rv PPIs. Analysis of sequence properties shows that, both host and pathogen proteins involved in host-pathogen PPIN tend to have longer primary sequences, tend to have more domains, tend to be more hydrophilic and tend to be less positively charged compared to other proteins in intra-species PPIN. Analysis of topological properties shows that not only host proteins but also pathogen proteins involved in the host-pathogen PPIN tend to be hubs in their own intra-species PPIN.

The prediction approach we discussed in this work has huge potential in applying to many other host-pathogen systems, and the properties that we have discovered through sequence and topological analyses may be helpful in understanding the host-pathogen PPIN and also provide alternative ways to assess predicted host-pathogen PPIN in a variety of different situations.

## Reviewers’ comments

We appreciate the reviewer’s comments from Prof Michael Gromiha, from Prof Narayanaswamy Srinivasan and from Prof Thomas Dandekar. We have revised the manuscript accordingly.

### Reviewer 1 (First Round): Prof Michael Gromiha, Dept of Biotechnology, IIT Madras

In this work the authors have proposed an accurate homology based prediction method for identifying host-pathogen interactions. The approach has been tested with *H. sapiens-M. tuberculosis* PPIS and showed that the results are promising. Further, the occurrence of charged residues have been discussed. The paper is well written and the results are presented in detail.

1. The definition for homology should be discussed in terms of sequence identity, coverage etc.

Authors’ response: *As we are using the BBH-LS software system for identifying homologous between different species, in the manuscript we use the definition of BBH-LS score threshold set as 0.01. As explained in our manuscript, BBH-LS uses a complex combination of sequence identity, coverage, and similarity of the genomic context to determine homology. So it is hard to give a straightforward definition. While it is possible to compute and provide sequence identity of the results determined at the BBH-LS score threshold of 0.01, doing so is very likely to mislead the readers on how the homologs were actually determined.*

2. For the analysis of protein sequence based properties, it will be better to report the statistical significance.

Authors’ response: *We have revised the manuscript by including the statistical significance by calculating the p-value based on Student’s t-test.*

3. In the title Proteint should be corrected into Protein. *Authors’ response: Thanks very much for pointing out the typo. We have revised the manuscript to get rid of the typo. We have changed the title of this manuscript into “Stringent Homology-based Prediction of H. sapiens-M. tuberculosis H37Rv Protein-Protein Interactions”*

### Reviewer 1 (Second Round): Prof Michael Gromiha, Dept of Biotechnology, IIT Madras

The authors addressed my comments.

Authors’ response: *Thanks very much for your comments and suggestions that made our work better.*

### Reviewer 2 (First Round): Prof Narayanaswamy Srinivasan, India Institute of Science

Authors aim to predict protein-protein interactions across human and mycobacterium tuberculosis primarily using homology with human and pathogen proteins respectively in a dataset of host-pathogen protein-protein interactions (PPIs). Use of a database with experimentally derived host-pathogen PPIs as a template to predict human-Mtb PPIs is the main feature proposed by authors as new in this manuscript.

Authors’ response: *Thanks very much for the comments.*

I would like to draw the attention of the authors to the paper Mulder NJ, Mazandu GK, Rapano HA (2013) Using Host-Pathogen Functional Interactions for Filtering Potential Drug Targets in Mycobacterium tuberculosis. J Mycobac Dis 3:126. doi: 10.4172/2161-1068.1000126. In this paper Mulder *et al.* have used PATRIC database (which is also used by the authors of current manuscript) to predict human - Mtb PPIs. Mulder *et al.* have also performed enrichment analysis. It is important that Zhou *et al.* compare their work with that of Mulder *et al.* and highlight the new and important points in the manuscript.

Authors’ response: *For the comments that Mulder et al. also use PATRIC, this may not be the case. We have read the paper very carefully and found that they predicted the human-mtb PPIs as follows, “Previously generated human and MTB intra-species functional networks were used. These functional networks were constructed by combining protein interaction data from the STRING database and complemented by additional interaction data from sequence and microarray data for the MTB network and by Bossi and Lehner’s interaction data, together with data from the REACTOME database for the human network, as depicted in Figure 1”. Obviously they are making the predictions using the different databases therefore this make the comparision less meaningful. In addition, we avoid referencing papers published by the OMICS Group. One of us (H. Zhou) actually just declined serving as an editor of the Journal “J Mycobac Dis” (where Mulder et al. published their work). The OMICS Group has the notorious reputation of producing some 250 journals without content and all of its journals charge high fee without any peer review. Refering to works on this journal may be harmful to the science community. As wikipedia says “An investigative report by The Chronicle of Higher Education stated that journal articles published by OMICS may undergo little or no peer review [*[59]*]. It was also suggested that OMICS provides lists of scientists as journal editors to create the impression of familiarity or scientific legitimacy, even though these are editors in name only and are not involved in the review or editing process [*[59]*]. Academics and the United States government, have questioned the validity of peer review by OMICS journals, the appropriateness of author fees and marketing, and the apparent advertising of the names of scientists as journal editors or conference speakers without their knowledge or permission. As a result, the U.S. National Institutes of Health no longer accepts OMICS publications for listing in PubMed Central and sent a cease-and-desist letter to OMICS in 2013, demanding that OMICS discontinue false claims of affiliation with U.S. government entities or employees”.*

Right from the beginning of the manuscript authors refer the proposed approach to host-pathogen PPI prediction as “accurate homology-based”. I appreciate the determination and enthusiasm of authors to achieve high accuracy in host-pathogen PPI prediction, However, I think claiming their method to be “accurate” almost as the name of the proposed method is inappropriate especially before the accuracy of the results obtained is demonstrated/proved beyond any doubt. Authors may more appropriately refer their method as “proposed method” or something like that. However I leave it to the discretion of the authors.

Authors’ response: *Thanks very much for the insightful comments. We have changed the title of this manuscript to avoid using the word “accurate”. Indeed, it is an excessive claim. We have changed the word “accurate” to “stringent” and change the title to “Stringent Homology-based Prediction of H. sapiens-M. tuberculosis H37Rv Protein-Protein Interactions”. And we have made this revision throughout the manuscript.*

Introduction section (page 3): Authors state “ii) the differences between prokaryotic and eukaryotic proteins are not considered.” It is not clear what are the differences between prokaryotic or eukaryotic proteins? Are there any general points here? Any reference to support author’s point?

Authors’ response: *The differences between prokaryotic or eukaryotic proteins have been reported in many papers and even classical text books. The major differences are listed in our manuscript already, post-transcriptional modifications, structures, etc. For the details of differences, please also refer to the following works, Nielsen et al. [*[60]*], Frye et al. [*[61]*], Chang et al. [*[62]*], Von et al. [*[63]*], von et al. [*[64]*], Kozak et al. [*[65]*], Hartley et al. [*[66]*], Springer et al. [*[67]*], Allfrey et al. [*[68]*], Neidhardt et al. [*[69]*], Schwartz et al. [*[70]*], Pestka et al. [*[71]*], Wallin et al. [*[72]*], Hartl et al. [*[73]*].*

In page page 4 authors mention post-translational modifications and structure. While I agree that post-translational modification is a difference between prokaryotic and eukaryotic proteins, it is not clear how realization of this difference helps in predicting human-prokaryote PPIs. I don’t think that structures of homologous proteins from prokaryotes and eukaryotes are radically different.

Authors’ response: *The differences in post-translational modification, protein structures, cleavage site, etc, may have influence in the interacting residues and interaction interfaces, which count a lot when transferring interactions from intra-species PPI to inter-species PPIs. Therefore, we made an improvement here in this work and it demonstrated a better performance.*

If the authors depend on using experimentally derived host-bacteria PPI database as the template to predict human-pathogen PPIs then comment, in the spirit of general applicability of the proposed approach, on 1. the limitation of the size of template dataset. 2. Completion and accuracy of the template dataset 3. prokaryote-dependent host-pathogen PPIs (i.e., if prokaryotes in the template and the target are very different, such as Gram negative and Gram positive, what is the specific advantage of using host-pathogen PPIs as the template?)

Authors’ response: *In the revised manuscript we discussed the limitation of the size, completion and accuracy of the template datasets. As currently the template datasets are very limited and we have already tried our best in finding the most abundant source of human-bacteria PPIs. The major limitation of our stringent homology-based approach lies in the fact that there is a limited amount of source eukaryote-prokaryote PPIs available currently. However, with the rapid advance in technology and the community’s increasing interest on host-microbe interaction studies, the eukaryote-prokaryote template PPIs will be much more abundant in the future. This should greatly facilitate the application of our stringent prediction approach to many host-pathogen systems in the future. It is a very insightful comment on the differences between the pathogens, say gram negative and gram positive. If the pathogens have drastic differences in their proteins (primary sequences, tertiary structure, interaction interfaces, and interacting residues, etc), then they will be less likely to be identified as stringent “homolog” in our approach as we are using the BBH-LS system. BBH-LS takes the origin and phylogenetics distances between two prokaryotes into account, as their genomic context will be calculated when identifying the homologs. Therefore if there are huge differences between one of the gram negative prokaryotic proteins and one of the gram positive prokaryotic proteins, they will unlikely to be reported as homologs in our stringent homology-based approach.*

Page 11: Paragraph under the section “Analysis of sequence properties of proteins involved in host-pathogen PPIs”. Authors seem to believe that sequence properties such as length, number of domains and degrees of domains will be different for proteins involved in intra-species interactions compared to those involved in inter-species interactions. What is the basis for this assertion? if this is correct what about proteins involved in both intra-species and inter-species interactions? Authors present some results on this in Tables 9 and 6. But the results are critically dependent on the accuracy and completeness of both predicted and experimentally determined inter-species and intra-species PPIs respectively. The main problem for me here is that I am unable to identify the scientific basis to expect differences in the sequence features of proteins involved in intra-species and inter-species interactions. I am also of the impression that only very small proportion of proteins are likely to be involved in exclusive intra or inter-species interactions. Most proteins (especially in the host) are likely to be involved in both inter and intra species interactions.

Authors’ response: *We are not assuming the sequence properties such as length, number of domains and degrees of domains will be different for proteins involved in intra-species interactions compared to those involved in inter-species interactions. On one hand, this section of analysis in the manuscript was just conducted to see if there is anything special for the proteins involved in the inter-species PPIN. From the results we get from the analysis, we are also surprised at the findings, but there is no assumption or assertion here in this section. We have simply discovered that those properties are different for the proteins involved in inter-species PPIN comparing with the proteins involved in intra-species PPIN. Sorry for the confusion, but the proteins we were conducting the analysis are exactly the proteins involved both in inter-species and intra-species PPIN, as long as the proteins involved in the inter-species PPIN, we will take them out and label them as proteins involved in inter-species PPIN. Any remaining proteins involved in intra-species PPIN will be labeled “proteins involved in intra-species PPIN.”*

Pages 15: Authors use the term “interaction strength” to refer the number of times the prediction of interaction between a host protein and a pathogen protein is made. Traditionally the term “interaction strength” refers to how tightly two proteins bind physically. Authors may want to use a more appropriate term such as “measure of reliability” or “consensus score”.

Authors’ response: *Thanks very much for the comments. We have revised the manuscript throughout, we have replaced the term “interaction strength” with “consensus score” to avoid the confusion.*

In page 15 authors claim that their proposed approach is more efficient than the conventional approach simply because their proposed approach predicts more number of interactions than the conventional approach. I feel this is inappropriate. I feel so because unless the accuracy of predicted interactions in the proposed approach is clearly quite high and is better than that of conventional approach it is inappropriate to refer it as “more efficient”. What in case much of the predicted interactions are wrong? Under such a circumstance there is no meaning to predicting higher number of interactions.

Authors’ response: *Here the term “efficient” is just describing the fact that stringent homology-based approach is using less templates but predicting more inter-species PPIs comparing with that of conventional homology-based approach. The evidence supporting the claim that our stringent homology-based approach is more accurate comparing with the conventional homology-based approach are listed in section “Cellular compartment distribution”, “Disease-related enrichment analysis”, “Functional enrichment analysis”, and “Pathway enrichment analysis”. All these results show that the human-mtb PPIN predicted by our stringent homology-based approach are more plausible, as they have more functional relevance to this pathogen’s infection.*

### Reviewer 2 (Second Round): Prof Narayanaswamy Srinivasan, India institute of Science

I do not want to discuss the reputation of a journal or a publishing group in this platform. However the article by Mulder NJ, Mazandu GK, and Rapano HA is a freely available document in the internet. Also a simple pubmed search shows a few other articles in this area by same or overlapping set of authors in other journals.

Authors’ response: *Thanks for the suggestion. We don’t wish to ignore the contribution of those authors to the community. But we also wish to avoid discussing of work from that journal.*

While I agree with the point that the proposed method is not very similar to that proposed by Mulder NJ, Mazandu GK and Rapano HA, “a right answer looks right whichever way you approach the problem” adding confidence to predictions made. I still feel it is important to address this point. However it is only my opinion and I leave it to the discretion of authors. Regarding author’s response to other comments I am OK with most of them. Though I do not entirely agree with authors on their analysis of sequence features of proteins involved in intra-species and inter-species interactions, I do not see it as a major problem. After all it is author’s paper - not mine!

Authors’ response: *Thanks very much for the appreciation of our effort both in the manuscript and in the revision, we are very grateful to your comments that made our work better. For the analysis of sequence features of the proteins both in inter- and intra-species PPIs, it is still a very initial and it hasn’t been attempted by other groups before. It still needs lots of improvements at the current stage, but we believe that reporting this analysis here in this work is very beneficial for other scientists in the field to follow up with similar analysis and also introduce improvements on this analysis. This may eventually lead to more exciting discoveries.*

### Reviewer 3 (First Round): Prof Thomas Dandekar, Biocenter, Am Hubland, University of Würzburg, Würzburg, Germany

Hufeng Zhou *et al.* report on accurate homology-based prediction of H.sapiens M.tuberculosis H37v proteint-protein interactions. Summary comments: - The paper presents a lot of data, applying in part techniques originating from the authors themselves, requiring to asses then again the performance of these techniques according to these earlier papers. Furthermore, the quality of the results needs to be assessed. - A major question is of course which of these predicted interactions do really happen in M.tuberculosis infection? In the view of this reviewer, the paper does not really answer these questions with sufficient clarity and certainty, so that the results, though a lot of different tables and interactions, are not yet really useful to the reader. Please revise the paper (major revision) according to the detailed comments below - then the power and impact of the paper will be much higher.

Authors’ response: *Thanks very much for the comments. We have revised the manuscript according to the reviewer’s comments and also provide a point to point reply listed below. According to our knowledge we have sufficiently assessed the results according to the latest technologies and available data allowed, although we do bear in mind that our validation is insufficient due to the missing of gold standard Human-M. Tuberculosis, and that is the limitation we realized and trying to improve in the future work on this project.*

Title “Accurate” is not what is delivered, we get lots of predictions, the whole approach is bound to get many over-predictions and detailed functional analysis of the predictions happens only at very few places in the manuscript. Currently a title such as “Abundant homology-based over-prediction of H.sapiens M.tuberculosis H37Rv potential protein-protein interactions by two different methods” would be more appropriate. Furthermore, already in the title is a typo, remove the t after “proteint”, otherwise this even more astonishes the reader in the context of “accurate”.

Authors’ response: *Thanks very much for the comments. We have revised the manuscript to get rid of the typo. We have changed the title of this manuscript to avoid using the word “accurate”, indeed, it is an excessive claim. We changed the word “accurate” into “stringent” and change the title into “Stringent Homology-based Prediction of H. sapiens-M. tuberculosis H37Rv Protein-Protein Interactions”*

Abstract: Should be adapted after revising the whole paper.

Authors’ response: *We have revised the abstract accordingly.*

Background: An important point and very useful to get a reasonable paper from your study is to define what you mean by “interaction”. This reviewer first assumed that you primarily wanted to predict a direct protein-protein interaction, in other words something that you can later directly experimentally verify, e.g., by immune precipitation, crosslink etc. If you instead just mean functional interaction, e.g. when you speak about receptor-hormone interactions involved in infection response or look at early and late gene expression in infection or effects on transcription factors then it is far more difficult for the reader to see, how far your list can help as of course there are far and close connections of such functional interactions and you never define how far then the functional interaction may still be and to what level of certainty you want to give your different interactions.

Authors’ response: *We highly appreciate the wonderful comments on the types of PPI. This should be clearly defined at the very beginning of the manuscript. As a matter of fact, we are actually predicting the direct physical interaction in a very stringent way, as the source database are primarily experimental physical interaction data and we use homology to stringently transfer the interaction data to the human-mtb system. In the revised manuscript we explicitly state this in the following words: “In this work, we only focus on the direct physical protein-protein interaction (PPI), therefore all the PPIs mentioned in this work are direct physical protein-protein interaction.”*

By the way, the papers you cite 7–11 are all from a bioinformatical “large-scale screen take it all” corner (Srinivasan group, Wuchty) it will significantly broaden the perspective if you include also some experimental papers which really delineate a host-pathogen interaction and the involved proteins - this then gives you also an opportunity to clarify which definition (direct or indirect, more functional protein-protein interaction) you want to follow more in the rest of your paper.

Authors’ response: *Thanks very much for the comments. We cite the works of Srinivasan et al., Wuchty et al. and so on (references 7–11) mainly because they are the representative work of conventional homology-based approach. Here, no matter conventional homology-based approach or stringent homology-based approach are all computational prediction approaches. In this work, we are predicting the direct physical interaction in a very stringent way, as the source database contains primarily experimental physical interaction data and we use homology to stringently transfer the interaction data to the human-mtb system. However, experimental approaches are out of the scope of this work.*

Methods Maybe call the first part “overview” so that the reader better understands what happens in the first paragraph.

Authors’ response: *The first part is called background, which is specified by the journal format requirement. We do not think we have liberty to change it.*

Prediction of host-pathogen PPI networks

Please remove the term “conventional homology-based prediction” as this suggests that this is the typical way to badly over-predict protein-protein interactions. Please remove the term “accurate homology-based prediction” as this suggests that this is the correct way to again grossly over predict physical protein-protein interactions between host and pathogen. Rather be neutral in both cases and call it according to what has really been done in both cases: intraspecies homology-based prediction instead of “conventional” and interspecies homology-based prediction instead of “accurate”. Furthermore, then the reader knows, both are computer-based homology assumptions and knows, ok, here are many over-predictions.

Authors’ response: *That is an insightful suggestion. However, using the new term of “intra-species homology-based prediction” and “interspecies homology-based prediction” may not be the best way of naming the different kinds of homology-based approach. In fact, it may make things worse: the naming may cause more confusion than convey a clear idea to the reader, as both homology-based prediction approaches we are discussing here are actually making inter-species PPI prediction. However, we have removed the word “accurate” and changed the “accurate homology-based ” prediction approach to “stringent homology-based” prediction approach.*

It may also be worthwhile to recheck if there is no large-scale M.tuberculosis interactome study available, so that you have a better basis for the first set of homology-based predictions. Similarly, there is a lot of literature from experiments available describing real and direct interactions during the course of infection with M.tuberculosis and it is these data that you should be really after if you want to predict with higher accuracy the real protein-protein interactions in the infection.

Authors’ response: *At the time of this work, we conducted a comprehensive work on the literature survey and was very sure there was no large-scale human-mtb interspecies host-pathogen PPIN available. It is true that intra-species large-scale M. tuberculosis interactomes are available with unknown quality (we did a comprehensive analysis on the available intra-species large-scale M. tuberculosis interactome, please refer to our BMC Genomics paper “Comparative analysis and assessment of M. tuberculosis H37Rv protein-protein interaction datasets”). However, our proposed “stringent homology-based prediction approach” only takes the inter-species eukaryote-prokaryote PPI data as the source PPI to make the predictions. Therefore, we are looking for large-scale human-mtb interspecies host-pathogen interactomes rather than rather than mtb intra-species interactome.*

p.6 BBH-LS is your own algorithm and it will be nice to the reader if you explain in a few sentences how it works, in particular how then sequence similarity is measured, by which algorithm, and how gene context is taken into account. Explaining this will also increase reader confidence into your large-scale data.

Authors’ response: *Thanks very much for the comments. It is really nice suggestions to explain more on the BBH-LS algorithm, and that will help to increase the reader confidence of our prediction approaches. However, BBH-LS algorithm is actually not our algorithm. The authors of BBH-LS developed their algorithms independently without any involvement from us. We get to know and used this algorithm through their publications on BMC system biology “BBH-LS: an algorithm for computing positional homologs using sequence and gene context similarity” by Zhang et al. But I strongly agree that BBH-LS algorithm needs to be explained more in our work to increase reader’s confidence. Therefore we have revised the manuscript accordingly “To identify the homologs between M. tuberculosis H37Rv and the 10 bacteria (in our stringent approach) and also the between M. tuberculosis H37Rv and H. sapiens (in the conventional approach), we use the BBH- LS algorithm which computes positional homologs using both sequence and gene context similarity [*[18]*]. BBH-LS is an effective and simple method to identify the positional homologs from the comparative analysis of two genomes, it integrates sequence similarity and gene context similarity in order to identify highly accurate orthologs [*[18]*]. This method applies the bidirectional best hit heuristic to a combination of sequence similarity and gene context similarity scores [*[18]*]. When applied BBH-LS algorith to the human, mouse, and rat genomes, it produced the best results when using both sequence and gene context information equally and compared to the other classic algorithms, (like MSOAR2) BBH-LS can identify more homologs with less false positives [*[18]*]. BBH-LS is considered to be a more accurate way of identifying homologs than other approaches which do not consider both the sequence and gene context similarity. The BBH-LS strength threshold β in this work is set as 0.01.”.*

p.9 PPIN - be so kind to remind the reader that this means protein-protein interaction networks

Authors’ response: *We have revised this part of manuscript accordingly. “Based on primary sequence analysis and topological analysis of the predicted host-pathogen protein-protein interaction network (PPIN).”*

p.9 Analysis of sequence properties of protein involved in the host-pathogen PPI.Here as well as in the corresponding parts of the results the rational behind this section remains dark. If I already have a list of over-predicted protein-protein interactions with many proteins only indirectly affected by the infection process,what do I learn from the domain or sequence property distribution in this? Would it help to understand that M.tuberculosis has a certain percentage of hydrophilic residues? The same applies to the domain lists. In the latter I agree that they can be interesting but only, if you spend more time in explaining and discussing their actual function and functional context for real examples in the PPI networks you found.

Authors’ response: *We utilized stringent homology-based approach accurately identifying homologs between mtb and other bacteria, and accurately transfer the experimental physical host-pathogen PPIs to predict the host-pathogen PPI in human-mtb system. Therefore, our results largely capture the possible human-mtb physical direct host-pathogen that most experimental approaches can detect. If experiments are applied in the mtb and human system, I believe all of our predicted human-mtb PPIs will be captured by the experiments. Moreover, the human proteins we analyzed in our human-mtb physical direct host-pathogen PPIs are actually the same human proteins involved in the human-bacteria inter-species PPIN from the source experimental data. Therefore we are analyzing the sequence and topological properties of an accurate dataset. The sequence and topological properties of host and pathogen proteins respectively as reported in this work, will be highly interesting and useful in this field of host-pathogen PPI studies, as those properties can used to differential the intra-species and inter-species PPIN, can be used in improving the prediction algorithms, and can be used in assessment and verification of predicted host-pathogen PPIs.*

p.10 Calderwood *et al.* is cited here as “the first” to analyze protein-protein interaction networks - please explain in what sense you think this is true, very probably Barabasi group was the first to analyze topological properties of interaction networks and such a citation should be given here or yours better explained.

Authors’ response: Calderwood et al. are the first to analyze the topological properties of the human proteins in host-pathogen PPIN, as thorough literature survey shows they coined this kind of analysis as early as 2009, and the second study conducted on the same analysis in 2012. Many groups and labs have reported the topological properties analysis results of *general* intra-species PPIN, not those involved in host-pathogen interactions. This is a subtle point. Nonetheless, we agree that claiming the Calderwood et al. work as the “the first” is not appropriate. We have revised this part of the manuscript accordingly.

Similarly, the statement “this work is the first-ever study that examines the intra-species PPIN topological properties” seems not convincing. You can cite work from the Guthke group (HKI Jena, Germany) just to have one concrete example of someone who did this before, but if you really check the literature I am sure there are other groups who already examined PPIN topologies between host and pathogen well before, for instance, if you think about the zig-zag model of Jones and Dangel in plant infection, this is also a topological description, right?

Authors’ response: *Thanks very much for the comments. Sorry for the confusion, we are not claiming ourselves to be the first of intra-species PPIN topological properties, but we are indeed the first to exam the intra-species PPIN topological properties of mtb protein involved in host-pathogen PPIN. That is, while there are many groups who work on intra-species PPIN topological properties before, no one before us has ever reported the intra-species PPIN topological properties of mtb protein involved in host-pathogen PPIN. Sorry for the unnecessary confusion, and we have also revised the manuscript accordingly on this part, by deleting the word “first”, although we are really the first on this small part of analysis.*

Results

Here again it is a good idea to phrase all results throughout more carefully, then even a more sceptic reader (in the moment my duty as one of the reviewers, my apologies) will be more convinced on the quality of the data presented. p.12 “... then the predicted results are solid.” - rephrase: “... then we can be more certain about the quality of our results” Unfortunately that is not at all the case for the poor reader, as you forgot to mention at the Background Section of the paper which type of PPI you want to predict. For instance if you want to predict direct physical interaction, then all compartments that have nothing to do with direct pathogen interaction of the host cell have to be completely removed from your prediction lists as they are clearly wrong predictions.

If on the other hand you just think of “functional connection” you should give some score for your prediction at the very least (for instance, you could choose either the different p-values or the different “interaction strength” you find in your calculations or both as such a decision score). A real sceptic would tend to say, leave the study all together because looser functional connections of the infection process are already very clear from the accumulated literature and it makes it only worse to assemble this in lists where you maybe leave some loos connected functions even OUT by your approach (opposite mistake, here mentioned for the first time by this referee in his comments and easy for you to check: Look at the gene expression data, there are many other genes which change during early or late infection, hence they are loosely connected to infection, but never turn up inyour selected list of homology-based predictions): So please, if you want to push the second direction, give a score on your different predictions to make them meaningful for the reader.

Authors’ response: *Thanks very much for the comments. I have revised the manuscript to clearly define the definition of PPI in our work, which is actually “direct physical interaction”, in the *Background* Section. Since we are not predicting functional associations, there is no need to give out the score of each association.*

The suggestion of removing compartments that has no obvious interaction with pathogen has to be discussed more carefully. (1) there may be many cellular compartments that actually interact with pathogens in reality but we do not yet know about them. For example, some research shows pile protein from E. coli may have interaction with proteins involved with apoptosis; therefore this pile protein interacts with human proteins that previously have no direct and obvious functional correlation until compiling experiments prove that. Especially at this stage of host-pathogen interaction studies, many things are still unknown; therefore we can not easily get a conclusion that some compartments that seem not obvious to directly interact with pathogens are wrong and needed to be removed. (2) Cellular compartment annotations are not complete and thorough. And some proteins may have been annotated with several terms. More over, some terms annotated to certain human proteins may not exactly indicate that these human proteins locate only in these compartments; it is also possible that these specific human proteins locate in some other cellular compartments as well. And we have also moderated some the sentences in the manuscript. For example, we have revised sentences like, p.12 “... then the predicted results are solid.” revised into “... then we can be more certain about the quality of our results”.

p.13 the “transcription factor complex” category is a good example for loosely connected interaction - clarify, give score if you want to mention such interactions, clearly remove if you are after direct physical interactions (then only true for a virus, there several of its proteins directly interact with transcription and translational machinery of the host, e.g. in the HIV example you mention).

Authors’ response: *See *Discussion* in the previous point.*

p.13., second part: “proteasome degredation” is believable, I fully agree and here you also really go after the functional connection of the interaction, bravo! If you would remove all over-predictions and only go for some protein-protein interactions new predicted which have a high probability to be direct host-pathogen interactions in infection or be functionally implied with a really high score, then you have achieved what your paper intends to be about: accurate host-pathogen protein-protein interaction prediction!

Authors’ response: *Thanks very much for the comments. Actually intend to predict direct physical interactions, and we start from the direct physical interaction of human-bacteria PPI to accurately infer the possible direct physical interaction between human and mtb.*

p.14, middle: comparison of your two homology-based approaches: After rephrasing them as suggested above, your statement becomes also more fair and makes also technically sense.

Authors’ response: *Thanks very much for the comments. We have revised this part of the manuscript to make the statement more modest and humble.*

p.14,15,16 including nuclear hormone receptors: To this reviewer this part seemed a superficial analysis, here the reader would need detailed analysis given from you to understand which interactions make really functional sense and are worth while checking experimentally. If you add this, then the paper becomes really useful and interesting. “nuclear hormone receptors regulate innate immunity response” - so here you tend to refer to functional interactions, so: give a comparative score, analyze individual interactions, and stress exactly those which are new, have not been reported for this interspecies PPI, and would be worthwhile to be pursued experimentally. This applies even more so for the intra-species homology-based approach.

Authors’ response: *Here in this part of the manuscript we are using the Functional enrichment analysis of proteins involved in direct physical host-pathogen PPIs. Although we are really predicting the direct physical interaction, in many cases, the real direct physical interactions are always correlated with strong functional basis. In other words, we are trying to assess our predicted direct physical interaction through the functional perspective, with the underline basis, “if the direct physical interactions are real, they will more or less have some functional relevance for the host-pathogen system.” Therefore, we are discussing in this part of manuscript in a way that, if we realize there are some evidence supporting the validity of the predicted host-pathogen direct physical interaction, we will add more discussion and evidence to further explain the predicted interactions. Here in this section of the manuscript, we are using the enrichment analysis of the targeted human Gene Ontology terms to achieve the goal of finding the possible functional correlation. Therefore, this is not a set of analysis that examining each PPI one by one, it is grouping all the targeted human proteins together as one set of proteins that involved in the direct physical interactions with the pathogen proteins, and if they are enriched in many functional terms closely related to the host-pathogen interaction more specifically the human-mtb interaction, the prediction of direct physical interaction as a whole dataset has desirable performance. Therefore in this section picking out the individual one PPI responsible to certain terms maybe not the correct way to explaining the validity of this work, as enrichment analysis is achieved by Hypergeometric test of the whole targeted human protein sets not one by one examining of each PPI.*

p. 17 “focal adhesion” - this is again a good example, nice result!

Authors’ response: *Thanks very much for the comments. Yeah, focal adhesion are among many terms that naturally show up in the final results, so overall our prediction results are very good.*

Discussion

Again, broaden the discussion and cite paper also in light of the above points: Cite some experimental verified interaction results and papers describing them, speak about different definitions to define PPI networks - and convince the reader more about the selected interactions you think are probably new and should be there.

Authors’ response: *There are limited work on the human-mtb direct physical PPIs available, and we haven’t found any experimentally verified human-mtb PPI simply because this kind of experimental verification are relatively few. But in the future work, we are thinking of experimentally verifing some of our predicted human-mtb PPIs.*

Cancer pathway discussion: A nice point, again in the discussion mention people who alerted the cancer community about this connection before (there are such cancer scientists who suggested this analogy with an infection before, but I agree, this is an exciting connection) and then again give a score to these observed functional interactions so that the reader knows which ones exactly to follow up.

Authors’ response: *Thanks very much for the comments. The cancer pathways showing up are really exciting here as they provide not only the new perspective of understanding the host-pathogen interaction, but also to some extent support our prediction of direct physical interactions. Here we are also doing the enrichment analysis, which are taking the whole set of the targeted human proteins as one set of human proteins involved in the interaction of human and mtb, and using the statistical approach to see which pathways might be significantly enriched for this set of human proteins. Therefore it has nothing to do the individual scoring of each functional association of the PPIs. Moreover we are actually predicting the direct physical interactions, pathway enrichment here are just an assessment approach for the predicted direct physical interactions from the functional aspect, as the underline premise is if the direct physical interactions are real, they are very likely to be supported by the functional point of view. Therefore we are not predicting the functional association but using the functional enrichment(no matter Gene Ontology or Pathways) to assess the validity of the predicted direct physical interaction.*

Hub proteins are more easily involved also in inter-species interactions: Fine, this makes sense. However, also here it would be nice that you really consider some concrete protein examples, then it becomes clear, why and how this specific hub proteins work within host or pathogen as well as in the interactome between them.

Authors’ response: *Thanks very much for the comments. It is relatively new topic for this field, around 3 years ago, one group first identified that human proteins involved in the host-pathogen PPIs tend to be the hubs of their own intra-species PPIN, and here we are the first to report this is also the true case for pathogen like mtb, as mtb proteins involved in host-pathogen PPIN also tend to be the hubs of the mtb intra-species PPIN. For the reason of why and how this is the case, why the proteins involved in the host-pathogen PPIN are more likely to be the hubs of their own inter-species PPIN are still under our heavy investigation, we are working very hard on explaining the theory and also supplemented with solid examples. But this will take a while for us to get the final conclusion. An in-depth discussion on topic is out of the scope of the current manuscript. Also, we can not include this part as we still don’t have a final answer why that is the case—but this is certainly worthwhile to report to the community so that many groups who are also interested in this topic can work on it and contribute to this study.*

Conclusion

Rewrite in light of all the comments: Key point what did you find and where you are so sure, that an experiment should confirm the homology-predicted novel interaction between host-pathogen proteins?

Authors’ response: *Thanks very much for the comments. We have revised the manuscript according to this review. Because of the assessment from localization, from functional enrichment and from pathway enrichment and also from the precise homology transformation, from the highly accurate human-bacteria source PPIs, we have the confidence that the data set we are predicting will be a suitable data set to start with when experimental verification is going to kick in.*

Tables, Figures: Figure 1: Very nice, that transmits a clear message! Please prune and reorder all results tables and figures, once you have decided whether you want to show direct physical interactions or all sorts of more or less direct functional interactions and how you then would score them.

Authors’ response: *We have revised the manuscript and clearly indicated that we are actually predicting the physical interaction at the beginning of the *Background* Section. And we have revised the manuscript throughout according to the comments of this reviewer, thanks very much for making our work better.*

### Reviewer 3 (Second Round): Prof Thomas Dandekar, Biocenter, Am Hubland, University of Würzburg, Würzburg, Germany

The authors made an effort to improve the manuscript. They kindly responded to the suggestions and points made, thank you, however, decided not to change much in the substance of the manuscript. Hence, I would ask the editor to reject the manuscript and in case of publication I strongly recommend to publish my comments so that the community is not mislead by overprediction and bad data.

Authors’ response: *We realize although our prediction performs better but we may still have some false positives in our prediction results which are very hard to avoid. We appreciate that, while our prediction is valuable, it is currently still a prediction and cannot be considered a golden standard yet. Only when all the predicted PPIs have gone through stringent experimental verification then we can claim that our prediction is real and can serve as a golden standard for the field.*

In case of publication the lowering of the impact factor by Biology Direct may perhaps not happen, as after publication against my strong advice this may become a good example on how to go wrong with confidence.

I appreciate that the authors did a lot of work, and the calculations are technically sound, but the net result achieved will be very misleading for any reader who really wants to know which proteins in M.tuberculosis physically or direct interact with the human host. Unless you do something drastic to your methods (e.g., include a scoring scheme for quality of interaction, consider the compartments of the proteins and whether they can interact actually under TB infection or look meticulously at the biological function) this will stay so.

Authors’ response: *We did provide the scoring scheme in this manuscript. It is called“consensus score”. This scoring scheme is primary the number of source PPI matches supporting the human-mtb PPI.*

For instance:

Titel: Simply cancel out “Stringent”, “accurate” etc. then it is clear for the reader

what you are doing: overprediction by two different methods of homology prediction.

Similarly “conventional” is also no “conventional” method but use the two terms “intraspecies homology-based prediction” as well as “interspecies homology-based prediction” instead of “stringent” or, previously “accurate” to not mislead the reader

Authors’ response: *As we discussed in the previous reply, intraspecies/interspecies homology-based prediction are also very confusing to the readers. Readers may be confused between what we are using to predict (source PPIs) and what we actually predict (predicted PPIs). For example, it can be confusing when we talked about “interspecies homology-based prediction to predict interspecies PPI”. It makes sense to propose the naming in this way, but it may cause more confusion. More importantly, our stringent homology-based prediction means more than interspecies homology-based prediction. For example, we are using human-bacteria source PPI to predict human-mtb PPIs. But if we are predicting human-archaea PPIs, we can not use common interspecies source PPIs like human-bacteria PPIs, we have to look for source human-archaea PPIs for this prediction. Therefore our stringent homology-based are very stringent on the type of proteins in the source PPIs and also stringent on the homology transfer, so that the homology prediction can achieve a better performance.*

Then, carefully concentrate on the big major point of my criticism in your study phrased already in the first round of reviewing: You now write “we only focus on the direct physical protein-protein interaction (PPI)” - very well intended, but unfortunately this is exactly not the case:You do a homology-based prediction in both cases.

Authors’ response: *There are several issues discussed in this section. First, we are predicting host-pathogen physical PPIs that is why we only transfer physical source PPIs in predicting the targeting host-pathogen PPI. Yes, we do homology-based prediction in both cases, but in both cases we are trying to predict physical host-pathogen PPIs, and in both cases we only use physical PPIs as the source PPIs to make prediction. Therefore we are predicting the direct physical interactions both in stringent and conventional approaches. We agree that it is possible that some of the predicted interactions may not be physical interactions. Nevertheless, some amount of false positives is unavoidable, and we believe (and the reviewer also agrees) our approach has many fewer false positives than earlier homology-based approaches.*

To check whether your protein-protein interaction by the - I agree - better method, the interspecies homologybased prediction, you need either a golden standard of experiments (not yet available for your example) or you have to meticulously check which of the numerous potential interactions have really to do with host-pathogen interaction in this specific organisms: human and M.tuberculosis. Some of the findings you discuss in these terms, but there is nothing systematic.

Authors’ response: *Limited by the current availability of data, especially for golden standard PPI datasets available for verification, we use indirect approaches to assess the performance of our prediction approach. We believe our effort on methodology will be very beneficial to the community.*

Another approach would be to really think about the infection process and then predict in which compartment proteins from the human host DIRECTLY see proteins of M.tuberculosis as you claim you are really after physical interactions. Currently most of the interactions I would consider to be functional and not direct, by pathway connection: the “transcription category” (p.16) is a good example for such loosely connected interaction (and previous criticism ignored), - clarify, give score if you want to mention such interactions, clearly remove if you are after direct physical interactions.

Authors’ response: *We are predicting direct physical interaction between host and pathogen proteins. We have discussed strong evidence that supports our predictions, as well as our claim that these predictions are more reliable than previous ones. The evidence includes the existence of homologous host-pathogen interactions, the evidence that the proteins involved are found in MTB infection-related pathways and compartments, etc. We have also provided a scoring scheme (the consensus score) to rank the predictions. We acknowledge that we do not have experimental data that directly verify the predicted physical interactions. But we hope that our predictions, which we believe are far more reliable than previous predictions, will be a useful guide to performing new experiments on human-MTB protein interactions.*

As already stated in my last round of comments, you need some scoring to be surer of which prediction to trust or not.

Authors’ response: *We have a scoring mechanism, which is called “consensus score”.*

Minor point:

Cancer pathway discussion: A nice point, again in the discussion mention people who alerted the cancer community about this connection before, I mean not the BMC Medical Genomics paper of 2009 but the basic concept that cancer is also sort of an infection the human host fights against.

Authors’ response: *thanks for pointing that out. We also believe cancer is sort of infection the human host fights against. In this paper, Coussens and Werb discussed a close relationship between infection and cancer [*[74]*]. We are also currently studying a virus (EBV), the infection of which will significantly increase the likelihood of cancer.*

There are more points to improve the manuscript, but my time is also limited.

Authors’ response: *Thanks for your time and help in improving our manuscript.*

## Competing interests

The authors declare that they have no competing interests.

## Authors’ contributions

This work was jointly conceived, planned, and written up by LW and HZ. The analytical experiments were performed by HZ and SG. HZ and NN using the BBH-LS to identify the homologs, HZ, JJ and MF designed the functional analysis, LZ, BL, MT, SL and GX offer critical suggestions on the protein hydrophilic analysis. All authors read and approved the final manuscript.

## Authors’ information

LW is a KITHCT chair professor in the School of Computing and a professor in the Yong Loo Lin School of Medicine at the National University of Singapore. Before that, he was the Deputy Executive Director for Research at A*STAR’s Institute for Infocomm Research. He currently works mostly on knowledge discovery technologies and their application to biomedicine.

HZ graduated from NUS Graduate School for Integrative Sciences & Engineering, and School of Computing, National University of Singapore, Singapore. He is currently working as research fellow at Department of Medicine, Brigham and Women’s Hospital, U.S.A, and postdoc research fellow at Department of Microbiology and Immunobiology, Harvard University, U.S.A.

NN and JJ are graduates student in School of Computing, National University of Singapore. HZ NN and JJ are lab mates stuying in the computational biology lab in School of Computing.

SG is a graduate student in Department of Environmental Health, Harvard School of Public Health, Harvard University, U.S.A. SG and HZ collaborated closely on this work in 2012 to 2013.

LZ is a graduate student in Bioinformatics Research Center, & School of Computer Engineering, Nanyang Technological University in Singapore. LZ was visiting the computational biology lab in School of Computing when this work was in progress.

BL is a postdoc at Computer Science Department, Carnegie Mellon University.

MT and SL are research fellows at Department of Medicine, Brigham and Women’s Hospital, U.S.A, and postdoc research fellow at Department of Microbiology and Immunobiology, Harvard University,U.S.A.

GX is a visiting graduate student in Department of Medicine, Brigham and Women’s Hospital, U.S.A.

## Supplementary Material

Additional file 1 **Predicted *H. sapiens-M. tuberculosis *H37Rv PPI datasets.** We predicted 1005 *H. sapiens-M. tuberculosis* H37Rv PPIs using the stringent homology-based prediction approach and 326 *H. sapiens-M. tuberculosis* H37Rv PPIs using the conventional homology-based prediction approach. All the PPI data are recorded in simple text format in this additional file. Click here for file

Additional file 2 **Results of GO enrichment analysis.** In Additional file 2 we put the complete results of GO enrichment analysis of *H. sapiens* proteins involved in the predicted *H. sapiens-M. tuberculosis* H37Rv PPIs. The GO terms includes all terms from level 1 to level 5 (threshold “count > 2, p-value <0.1”). Click here for file

Additional file 3 **Cellular compartment distribution of the *H. sapiens *proteins targeted in the predicted *H. sapiens-M. tuberculosis *H37Rv PPI datasets.** We calculated the Cellular compartment distribution of *H. sapiens* proteins targeted in the predicted *H. sapiens-M. tuberculosis* H37Rv PPI datasets. The two predicted Host-Pathogen PPI datasets are predicted by the stringent homology-based approach and the conventional homology-based approach. Click here for file
